# Stem Cell Differentiation Disperses Transcriptional Clusters via a Conserved Surface‐Condensate Trajectory

**DOI:** 10.1002/advs.75924

**Published:** 2026-06-09

**Authors:** Tim Klingberg, Irina Wachter, Agnieszka Pancholi, Matthias Akyel, Yomna Gohar, Priya Kumar, Ana Miguel Fernandes, Yuzhi Bao, Alica Schmidt‐Heydt, Marcel Piepers, Alicia Günthel, Marcel Sobucki, Elisa Kämmer, Süheyla Eroğlu‐Kayıkçı, Stephan Allgeier, Sylvia Erhardt, Vasily Zaburdaev, Carmelo Ferrai, Lennart Hilbert

**Affiliations:** ^1^ Department of Biology Friedrich‐Alexander‐Universität Erlangen‐Nürnberg Erlangen Germany; ^2^ Max‐Planck‐Zentrum für Physik und Medizin Erlangen Germany; ^3^ Institute of Biological and Chemical Systems Karlsruhe Institute of Technology Eggenstein‐Leopoldshafen Germany; ^4^ Zoological Institute Karlsruhe Institute of Technology Karlsruhe Germany; ^5^ Institute of Pathology University Medical Center Göttingen Göttingen Germany; ^6^ Max‐Delbrück‐Center for Molecular Medicine in the Helmholtz Association Berlin Institute for Medical Systems Biology Berlin Germany; ^7^ Humboldt‐Universität zu Berlin Berlin Germany; ^8^ KASTEL — Institute of Information Security and Dependability Karlsruhe Institute of Technology Karlsruhe Germany; ^9^ Institute for Automation and Applied Informatics Karlsruhe Institute of Technology Eggenstein‐Leopoldshafen Germany

**Keywords:** biomolecular condensates, enhancer transcription control, gene expression, nuclear organization, stem cell differentiation

## Abstract

Stem cells exhibit exceptionally prominent transcriptional clusters, which dissolve with progressing differentiation. Although these clusters are assigned central roles in embryonic gene regulation, their formation and loss during differentiation remain poorly understood. This study reveals that these prominent clusters disperse along a conserved trajectory in mouse embryonic stem cells, fruit fly testes, and zebrafish embryos. Imaging and lattice simulations show that these clusters form via surface condensation on H3K27ac‐marked super‐enhancer regions, which act as genomic scaffolds. Upon differentiation, partial loss of these active epigenetic marks and transcription‐driven unfolding lead to dispersal of the prominent clusters. The block copolymer‐based lattice simulations explain this process as a conserved trajectory through a three‐dimensional state space, governed by surface condensation principles that extend beyond canonical liquid–liquid phase separation. This work marks surface condensation as a biophysical mechanism for the dynamic organization of stem cell‐specific transcriptional hubs and demonstrates evolutionary conservation in several organisms. By uncovering a conserved biophysical mechanism for transcriptional organization in development, our work illustrates how polymer properties can contribute to the control of cell identity and fate.

## Graphical abstract

Stem cell differentiation follows a conserved surface condensate trajectory: H3K27ac super enhancers nucleate large RNA polymerase II clusters that grow and unfold before transcriptional activity disperses them. This work reveals how biophysical forces at enhancer surfaces dynamically build and dismantle stem cell transcription hubs, reshaping cell identity across species.

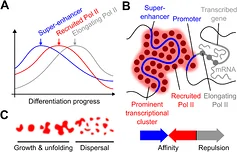

## 1 Introduction

The control over the selective transcription of genes into RNA in stem cells of the developing embryo faces different requirements than in the differentiated cells of a fully grown organism. These requirements are mirrored in distinctive features of the organization of the transcriptional machinery in stem cells [1]. One of these features consists of regulatory contacts that connect distal enhancers to the promoters of extensive sets of target genes over unusually long ranges (≈1 Megabase and above). These long‐range interactions often cross boundaries between topologically associating domains (TADs) and rely on protein clustering rather than cohesin‐mediated looping. Specifically, genes were proposed to associate with stem cell‐specific, prominent transcriptional clusters. Such prominent clusters have been observed in human cells [2, 3, 4, 5, 6, 7, 8, 9, 10], but also in models of mammalian pluripotency, such as mouse embryonic stem cells (mESCs) [11, 12], as well as models of embryonic development, such as Drosophila (fruit fly) [13, 14, 15] or zebrafish [16, 17, 18, 19, 20, 21]. Earlier works proposed the concept of transcription factories [3, 4, 10, 22, 23], which is continuously debated and appears related to some organizational and regulatory properties also of stem cell‐specific transcriptional clusters [24]. These clusters spatially concentrate transcriptional regulators, such as Mediator and BRD4, and the core transcription machinery, crucially the RNA polymerase II (Pol II) complex responsible for the transcription of most eukaryotic genes. Turning specifically to Pol II, clusters observed in differentiated cells persist only for approximately 10 s and contain only several tens of Pol II complexes [6, 25, 26]. In pluripotent cells, additionally, exceptionally prominent Pol II clusters are found, which persist for tens of minutes and contain hundreds of Pol II complexes [12, 19]. In mESC cultures, the loss of such prominent clusters was observed upon experimentally induced differentiation, suggesting a gradual transition from the stem cell‐specific configuration with prominent clusters to only short‐lived and smaller clusters upon differentiation [12]. However, whether the loss of such prominent Pol II clusters upon stem cell differentiation is a conserved feature across different organisms remains largely unexplored.

To conceptualize how the clustering of Pol II and transcription factors associated with different steps of transcriptional control might emerge, a combination of liquid‐phase properties and enhancer‐associated epigenetic chromatin marks has been proposed as a hypothetical mechanism [12, 27, 28, 29, 30, 31, 32, 33]. How could these mechanisms be applied to super‐enhancers, which are frequently associated with cellular pluripotency and are central to developmental transcription control [34, 35, 36, 37]? A current working hypothesis is provided via a surface‐condensation model, which combines both mechanisms to conceptualize the formation of super‐enhancer‐associated condensates via a surface condensation process [1, 19, 38, 39, 40]. Differently from canonical liquid–liquid phase separation (LLPS) models, which result from homotypic interactions among the phase‐separating species, surface condensation additionally requires interactions directed toward surfaces—for example super‐enhancers with the epigenetic H3K27ac mark. Due to these surface‐directed interactions, surface condensates can form at concentrations below the saturation concentration ($c_{sat}$) required for canonical (LLPS) [1, 19, 38, 39, 40]. In this subsaturated regime, a limit on the growth of surface condensates is set by the number of binding sites provided by a given surface, resulting in condensates at length scales matching their supporting chromatin regions. This growth limit contrasts with canonical LLPS, where over time the distribution of the phase‐separating species coarsens into a single droplet, whose size is limited by the available amount of the phase‐separating species. Growth limitation to relevant condensate sizes, explainability of condensate formation at subsaturated concentrations, condensate association with specific chromatin regions, and the clearly described mechanistic complementarity of homotypic interactions and chromatin‐directed interactions mark the surface condensation model as an intriguing concept for the understanding of transcriptional condensates.

The phosphorylation of the Pol II subunit 1 C‐terminal domain (CTD) plays a central role in the formation of super‐enhancer‐associated clusters [19]. The Pol II CTD phosphorylation marks undergo precise modulation during embryonic development and differentiation of stem cells, underscoring their potential importance in the formation of stem cell‐specific Pol II clusters [41, 42, 43, 44, 45]. The canonical function of Pol II subunit 1 CTD phosphorylation is the control of Pol II transcriptional activity and related molecular processes. Crucially, Pol II CTD phosphorylation additionally modulates liquid‐phase affinities of Pol II, leading to localization of Pol II with different CTD phosphorylation states into distinct, but adjacently placed spatial compartments on the scale of approximately 100 nm [19, 46, 47, 48, 49, 50]. A first key control step consists in the phosphorylation of the Pol II CTD heptapeptide repeat sequence (N‐Tyr1‐Ser2‐Pro3‐Thr4‐Ser5‐Pro6‐Ser7‐C) at the serine 5 position by cyclin‐dependent kinase 7 (CDK7). This serine 5 phosphorylation occurs during the initial recruitment of Pol II to gene promoters, during which Pol II forms dynamic macromolecular clusters [6, 25, 51]. A large fraction of the recruited Pol II is released while retaining the Ser5P mark, acting as a diffusible species with liquid‐phase affinity for transcriptional condensates [19, 52, 53, 54]. In stem cells, this recruited but diffusible form of Pol II contributes as an integral part to the super‐enhancer‐associated, prominent clusters that form in line with the biophysical process of surface condensation [12, 19].

To transition from the recruited to the elongating state, the CTD of promoter‐associated Pol II undergoes a second key control step, requiring the additional phosphorylation at the CTD repeat serine 2 position by cyclin‐dependent kinase 9 (CDK9). The canonical function of CDK9‐mediated phosphorylation is to release Pol II from its promoter‐proximal position into the gene body for transcript elongation. Additionally, this second CTD phosphorylation modifies the Pol II liquid‐phase affinity, resulting in its incompatibility with the condensates containing recruited Pol II [19, 29, 47]. In differentiated cells, this change in affinity is reflected as genes bearing elongating Pol II translocate from recruitment‐associated transcriptional clusters to distinct compartments associated with RNA splicing [29, 55, 56]. In line with this phase incompatibility, transcriptional activation of genes also exerts a dispersing effect, destabilizing transcriptional clusters [25, 57]. Some embryonically expressed genes undergo transcriptional activation specifically via the association with the prominent clusters enriched in recruited Pol II [20]. Upon transcriptional activation, these genes are repelled from the Pol II clusters and exert an effect similar to an amphiphile, thereby unfolding or even splitting apart these clusters [19, 58]. These observations represent a dispersing effect of transcribed genes that, combined with the surface condensation on super‐enhancers, defines a biophysical model for the formation and shape of the stem cell‐specific prominent Pol II clusters [1].

Given that prominent Pol II clusters occur in several stem cell and embryo systems and that their formation can be traced back to surface condensation as a biophysical model, we hypothesize that their loss is a conserved phenomenon across various scenarios of differentiation. In particular, we would expect that changes in the shape and ultimately the loss of prominent Pol II clusters over the course of differentiation should be consistently coordinated with marked changes in Pol II CTD phosphorylation and super‐enhancer activity. Additionally, if the biophysical surface condensate model applies not only in the stem cell state but also during differentiation, these changes in shape should be predictable from this model. To critically test our hypothesis, we first assess three different experimental models of stem cell differentiation: neuronal fate induction in mESC, sperm precursor formation in fruit fly testes, and germ layer induction in zebrafish embryos. In all cases, we find that differentiation is accompanied by a reduction in numbers of prominent Pol II clusters. During differentiation, we observe transient increases first in recruited and then in elongating Pol II, which are closely coordinated with initial rounding up and subsequent unfolding and dispersal of prominent Pol II clusters. We then confirm that differentiation and loss of prominent Pol II clusters are accompanied by a successive reduction in the active enhancer mark histone 3 lysine 27 acetylation (H3K27ac), indicating a loss of super‐enhancers. We proceed to formulate a block copolymer implementation of the surface‐condensation model on a two‐dimensional simulation lattice, which matches the changes in shape of Pol II clusters over five stages of early zebrafish development. Lastly, we chemically perturb super‐enhancer state and Pol II pause‐release, confirming surface condensation and transcription‐mediated dispersal as causal mechanisms in experiments as well as in the lattice simulations. Differentiation thus implies a stereotypic surface‐condensate trajectory, with transient increases in recruited and then elongating Pol II preceding the unfolding, spreading, and eventual loss of clusters associated with H3K27ac‐marked super‐enhancers. Together, these observations indicate that changes in super‐enhancer scaffolds and Pol II phosphorylation are sufficient to dismantle stem cell‐specific transcriptional hubs and couple the dynamic assembly of prominent Pol II clusters to major transitions in cell identity.

## 2 Results

### 2.1 Prominent Pol II Clusters Change Shape and Subsequently Disperse During Early Neuronal Fate Induction

**FIGURE 1:**
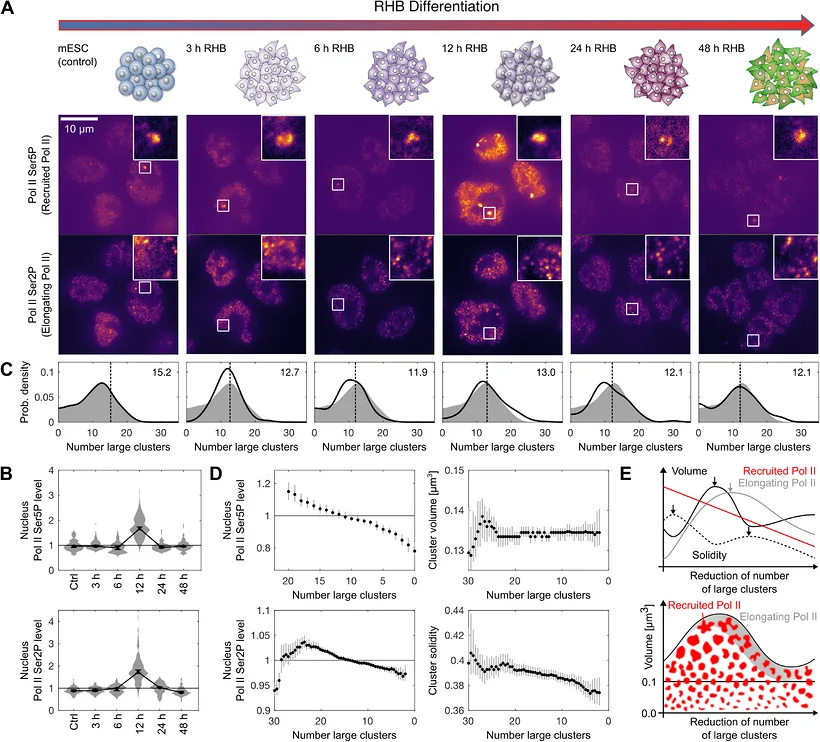
Change in shape and subsequent dispersal of prominent Pol II clusters in mESC upon induced differentiation. (A) Neuronal differentiation was induced with an RHB‐A medium and samples were collected over several time points after induction. Example micrographs of mESC nuclei with Pol II Ser5P (recruited Pol II) and Pol II Ser2P (elongating Pol II) labeled by immunofluorescence. Single optical sections obtained by instant‐SIM confocal microscopy. (B) Whole‐nucleus immunofluorescence intensities, normalized against the median of all values, data points are median with 95% bootstrap confidence interval. For the different conditions, n=105,119,101,177,140,99 nuclei from two independent experimental repeats were detected. (C) Distributions of counts of prominent Pol II clusters (detection in the Pol II Ser5P channel, cluster volume threshold $0.1\;\mu m^{3}$) per nucleus. Kernel‐based empirical probability density distribution, dashed line and text indicating arithmetic mean, the gray area indicates the distribution in the control condition for reference, the axis range was limited to 35 and omits outliers above this value. (D) Nuclei are binned based on a sliding window for the number of prominent Pol II clusters per nucleus to calculate for a given bin the nuclear Pol II Ser5P and Pol II Ser2P intensity. Further, all prominent Pol II clusters ($V\geq 0.1\;\mu m^{3}$) from all nuclei in a given bin are used to calculate cluster volume and solidity (median with 95% bootstrap confidence interval). 741 nuclei and 9496 clusters included in total. Running window with median and 95% bootstrap confidence interval. Running window parameters: window width 12, minimal number of data points 12. (E) Summary sketch explaining the shape transition leading to loss of large clusters.

We aimed to monitor the main steps of transcription control as well as the suspected loss of prominent transcriptional clusters in different models of cell fate induction. To this end, we marked different serine phosphorylation patterns of the Pol II subunit 1 CTD using multi‐color immunofluorescence (Figure 1A). In particular, recruited Pol II is detected via the phosphorylation of serine 5 of the CTD repeat (Pol II Ser5P). Elongating Pol II is detected via the additional phosphorylation of the serine 2 (Pol II Ser2P). As shown previously, indirect immunofluorescence detection of Pol II Ser5P and Pol II Ser2P serves to reliably visualize and distinguish the in situ levels and spatial distribution of recruited Pol II and elongating Pol II, respectively [16, 19]. Note that the correspondence of immunofluorescence detection of the Pol II phosphorylation marks in high‐resolution imaging with live‐sample detection via phosphorylation‐specific antibody fragments has been previously validated [19]. Throughout this study, we used instant structured illumination microscopy (instant‐SIM) [59], which combines the ability of super‐resolved visualization of the distribution of Pol II Ser5P and Pol II Ser2P fluorescence signal with sufficient experimental throughput to allow assessment of multiple experimental model systems. Instant‐SIM revealed several prominent clusters of recruited Pol II in mESC cultures (Figure 1A), mirroring observations from previous work [12]. Given the broad evolutionary conservation of the Pol II heptad repeat motif, this immunofluorescence‐based visualization can be applied to different eukaryotic model systems.

The first experimental model we assessed was the previously documented loss of prominent clusters of recruited Pol II upon differentiation of mESC cultures [12]. Here, we induced neuronal fate by RHB‐A cell culture medium, a protocol that robustly commits mESCs to a rapid pluripotency exit toward neuronal fate [45, 60]. A rapid pluripotency exit and neuronal‐fate‐specific transcriptional response of mESC cultures to this protocol were confirmed by the quick drop in transcripts of the pluripotency marker Nanog already at 12 h, together with Oct4 transcripts decaying from 12 h and being largely abolished at 72 h. Concomitantly, neuronal markers Blbp and Hes5 became transcribed at 72 h, showing efficient commitment toward neuronal fate (Figure S1A,B). Visual inspection of microscopy data suggested that these early steps in cell fate choice are accompanied by a systematic and transient increase in the nucleus‐wide levels of recruited and elongating Pol II (Figure 1A). Indeed, a transient increase in the nucleus‐wide level of recruited Pol II occurred specifically at 12 h (Figure 1B). The nucleus‐wide level of elongating Pol II also reached a maximum at 12 h, partially decayed at 24 h, and returned back to initial levels at 48 h (Figure 1B). Assessment by Western Blot confirmed the peak in Pol II CTD serine 5 phosphorylation at 12 h and showed that overall levels of Pol II were constant, attributing the observed peak to changes in CTD phosphorylation of a stable pool of Pol II (Figure 1C,D). An analysis of the fraction of mitotic cells over the differentiation time course showed no clear changes, ruling out the possibility that the sharply timed response at 12 h results from cell cycle or cell division synchronization as a main underlying cause (Figure S5). These observations reveal a transient transcriptional activation occurring around 12 h following RHB‐A induction, which precedes the transcription of neuronal fate marker genes at later time points and is sufficiently broad to transiently shift a significant fraction of the global pool of Pol II into recruitment and elongation.

Closer inspection of the prominent clusters of recruited Pol II in our microscopy images suggested a transient rounding up and volume increase around the 12 h time point (Figure 1A, zoomed‐in views). Also the levels of elongating Pol II in the vicinity of the large clusters appeared to change, indicating changes in the transcription activity of genes associated with these clusters (Figure 1A, zoomed‐in views). To consolidate these impressions, we applied a 3D object segmentation algorithm to the Pol II Ser5P channel, using a minimum volume threshold of $0.1\;\mu m^{3}$ to select large clusters for downstream analysis (Figure S2). Based on this analysis, from here on we refer to these large clusters of recruited Pol II, detected in the Pol II Ser5P image channel, simply as prominent Pol II clusters. As a first analysis step, we counted the number of prominent Pol II clusters per cell nucleus to assess the loss of large clusters over the differentiation time course. The number of prominent Pol II clusters per nucleus decreased from ≈15 to ≈12 by 6 h (Figure S3A), indicating a complex relationship between this early reduction in the number of large clusters and the changes in transcriptional control at 12 h. Additionally, the number of large clusters per nucleus displayed a wide distribution ranging from 0 up to ≈30 clusters (Figure 1C). The gradual loss of large clusters by 6 h, the total number of large clusters per cell, and the observation of a wide distribution of numbers of large clusters agree with previous work [12]. Also, the nucleus‐wide levels of recruited and elongating Pol II exhibited a broad distribution at the single‐cell level (Figure 1B). We interpreted these wide distributions as reflecting the cell population heterogeneity known for mESC differentiation procedures and aimed to stratify this heterogeneity to better understand the apparent complex relationship between the reduction of the number of large clusters and transcription control. To this end, we pooled all time points and sorted the single‐cell results using the number of prominent Pol II clusters per nucleus as a surrogate coordinate (Figure 1D). After this sorting step, the level of recruited Pol II decreased progressively along with the decreasing number of large Pol II clusters, suggesting a successful stratification of our single‐cell data (Figure 1D). The sorting also revealed a transient increase in the level of elongating Pol II at the prominent Pol II clusters with a peak at ≈23 prominent Pol II clusters per nucleus, suggesting a transient transcriptional response to the neuronal cell fate induction (Figure 1D).

To complete our analysis, we formally tested our visual impression that the prominent Pol II clusters undergo shape changes. To characterize cluster shape, we extracted the prominent Pol II clusters' volume as well as their solidity, a geometric shape descriptor that quantifies how “rounded” the clusters are. Greater cluster volume was previously attributed to an increased amount of H3K27ac‐marked chromatin within a given cluster and globally elevated levels of recruited Pol II, whereas lower solidity resulted from the presence of transcribed genes in the vicinity of clusters and disruption of the liquid‐phase affinity of recruited Pol II [19, 58]. Cluster volume was increased specifically at 12 h, coinciding with the transient increase of recruited and elongating Pol II at this time point (Figure S3A). Cluster solidity increased and decreased repeatedly over the differentiation time points (Figure S3A), indicating complex and interacting changes in cluster volume and shape (see Figure S3B for two‐dimensional volume‐solidity plots). To clarify the coordination between transcriptional control, cluster shape, and reduction in cluster number, we again used the number of large clusters per nucleus as a surrogate coordinate. In this representation, Pol II clusters transiently increase in volume, with a peak at ≈26 prominent clusters per nucleus and a slight rebound toward higher volumes at ≈20 clusters per nucleus and less (Figure 1D). The solidity decreased from an initially high value at ≈30 large clusters toward a minimum that coincides with the volume peak at ≈26, followed by a transient peak at ≈20 clusters per nucleus (Figure 1D). To rule out that our results are specific to the RHB‐A medium protocol and to test the robustness of our analysis approach, we repeated all steps for a different differentiation protocol, withdrawal of leukemia inhibitory factor (LIF). The changes in the number of clusters per nucleus and Pol II phosphorylation over the differentiation time points were similar, whereas volume and solidity changes differed from those obtained for the RHB‐A protocol (Figure S3C,D). When using the number of large clusters per nucleus as a surrogate coordinate, however, results from the LIF protocol were highly comparable to those from the RHB‐A protocol (Figure S4). This comparison supports the robustness of using the number of large clusters per nucleus as a surrogate coordinate and strengthens the conclusions obtained with the RHB‐A protocol. To make these results easier to interpret, we combined them into an explanatory sketch (Figure 1E). This sketch illustrates how the prominent Pol II clusters typical of the stem cell state initially round up and increase in size, then unfold as transcript elongation increases and size increases even more, and finally disperse into smaller clusters as differentiation progresses. The final decrease in solidity can be attributed to only unfolded clusters remaining above the volume threshold.

### 2.2 Change of Shape and Dispersal of Pol II Clusters are Conserved in Sperm Cell Precursor Formation

**FIGURE 2:**
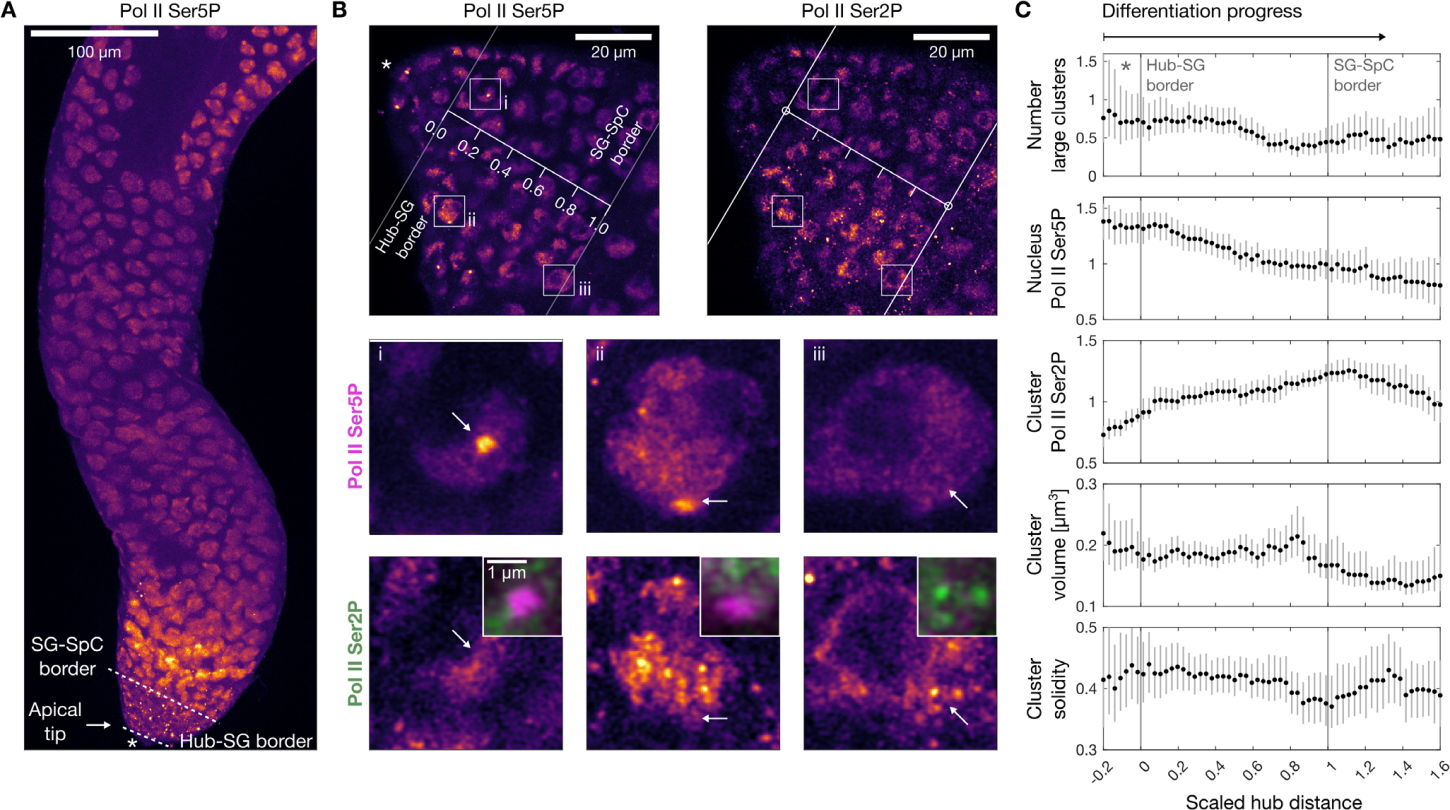
Change in shape and subsequent dispersal of prominent Pol II clusters during sperm precursor formation in fruit fly testes. (A) Representative single optical section of a fruit fly testis. Apical tip, hub‐spermatogonia (Hub‐SG) border, and spermatogonia‐spermatocyte (SG‐SpC) border are indicated. (B) Apical tip, the coordinate value 0.0 corresponds to the Hub‐SG border and the value 1.0 corresponds to the SG‐SpC border, providing a scaled differentiation coordinate (placed by visual assessment). Volumetric data were acquired using an LSM 900 confocal microscope with Airyscan 2 in super‐resolution imaging mode, obtaining resolutions comparable to the instant‐SIM data (see Methods). Detail views show nuclei with large compacted (i), large unfolded (ii), and small (iii) recruited Pol II clusters, and their spatial relationship to elongating Pol II. (C) Running window analysis of number of prominent Pol II clusters ($V\geq 0.1\;\mu m^{3}$) per nucleus, nucleus Pol II Ser5P intensities (normalized by median of all nuclei pooled), Pol II Ser2P intensities of prominent Pol II clusters, cluster volume, and cluster solidity along the scaled coordinate. Median with 95% bootstrap confidence interval. Overall, 218 clusters were analyzed from a total of 380 nuclei obtained from 10 samples from 3 independent experiments.

To assess progressing differentiation of stem cells also in a developed organ instead of a laboratory cell culture system, we investigated the formation of sperm precursor cells in the fruit fly. These cells are formed by the differentiation of germ line stem cells, which are positioned in a hub at the apical tip of a testis (Figure 2A) [61]. An increasing distance from the apical tip can be used as an indicator of the degree of differentiation, providing a coordinate to sort cells by their degree of differentiation that does not rely on computational sorting (Figure 2B). Along this coordinate, levels of recruited and elongating Pol II seem to change systematically (Figure 2B). Also, prominent clusters of recruited Pol II are apparent, which seem to undergo changes in size and shape along the differentiation coordinate (Figure 2B, zoomed‐in views). Using the same analysis as for mESC cultures, we find, on average, less than one prominent Pol II cluster per nucleus (Figure 2C). The frequency of occurrence decreases continuously with differentiation, confirming a gradual loss of prominent Pol II clusters (Figure 2C). As seen for mESC differentiation, nucleus‐wide levels of recruited Pol II decrease continuously and levels of elongating Pol II in the vicinity of the prominent clusters exhibit a transient peak, around a coordinate value of ≈1.1 (Figure 2C). Equally, cluster volume peaks before elongating Pol II (coordinate value of ≈0.8, Figure 2C). The solidity exhibited an early peak at ≈0.0 and a second peak located at ≈1.3, after the peak in elongating Pol II (Figure 2C). A combined analysis of volume and solidity mirrors the sliding window analysis, revealing two peaks in the ratio of high‐solidity over low‐solidity large clusters, first at hub distances [−0.2,0.2] and at hub distances [1.2,1.6] (Figure S7A). A more detailed analysis reveals that, specifically for large, low‐solidity clusters of recruited Pol II, spots of elongating Pol II are located more closely (Figure S7B), confirming that this previously characterized correlation also occurs in this model system [19]. The observation of only 1‐2 prominent Pol II clusters contrasts with up to ≈20 prominent clusters in mESC and might result from the specific transcription profile of hub cells and early spermatogonia. Their relatively focused, niche‐ and early‐lineage‐specific transcriptional program contrasts with the broader, spermatocyte‐specific gene activation [62]. This focused program might favor accumulation of Pol II into a few or even a single large cluster. Further, super‐resolution imaging of spermatocytes revealed that, as differentiation proceeds, Y loops containing nucleosome clutches and decorated by multiple smaller Pol II clusters emerge, marking a transition to multiple, smaller transcriptional clusters [63]. Taken together, the loss of prominent Pol II clusters, changes in Pol II modifications, and changes in cluster volume and solidity during sperm cell precursor formation match the observations during the differentiation of mESCs. This alignment of observations indicates a conserved sequence of change in shape and subsequent dispersal of prominent Pol II clusters during the differentiation of stem cells emanating from a stem cell niche of an adult animal.

### 2.3 Change in Shape and Dispersal of Pol II Clusters are Conserved in Germ Layer Induction in Zebrafish Embryos

**FIGURE 3:**
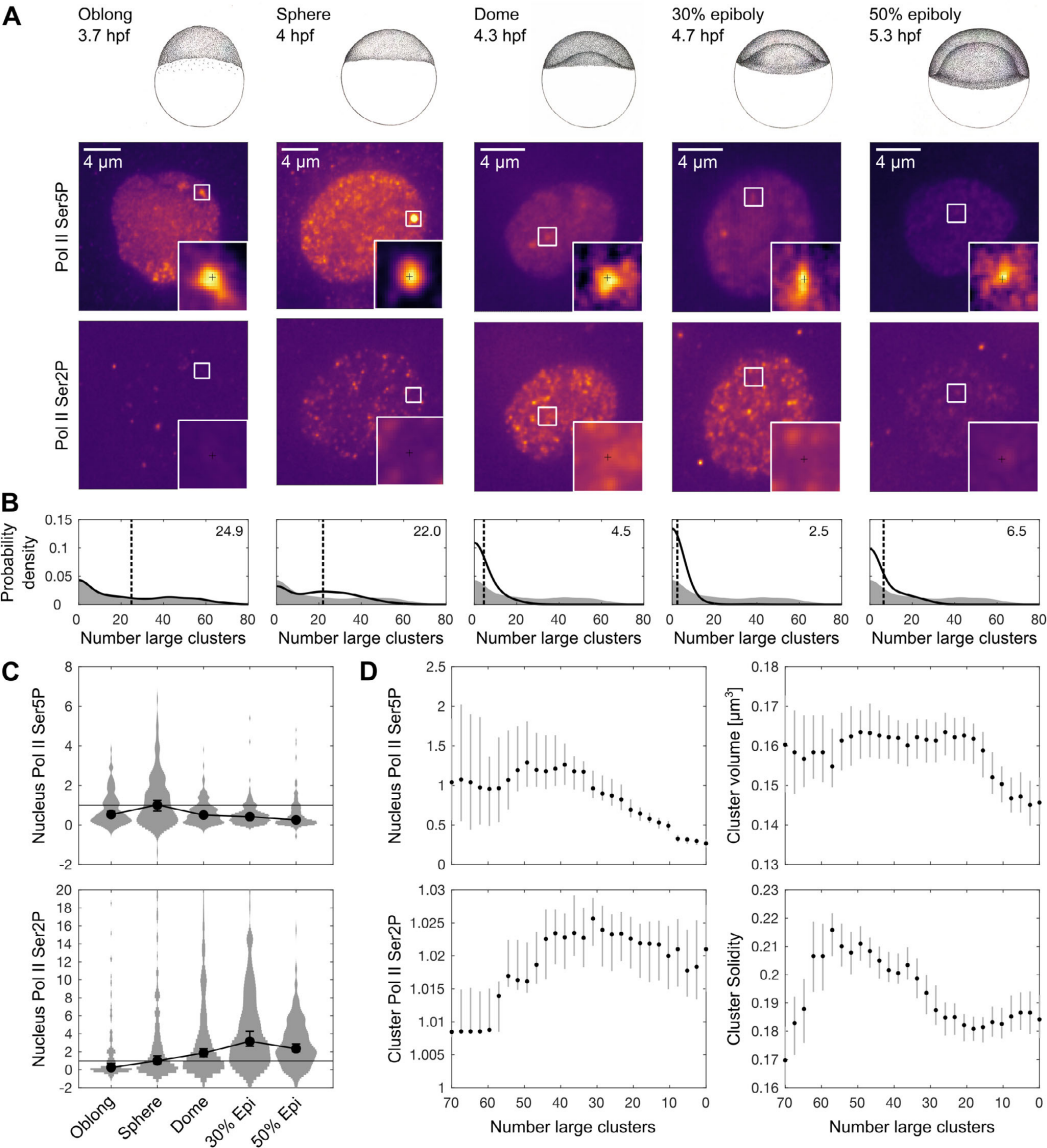
Change in shape and subsequent dispersal of prominent Pol II clusters during germ layer induction in zebrafish embryos. (A) Illustration of a zebrafish embryo approaching full transcriptional activation (oblong stage), reaching pluripotency (sphere stage), and undergoing progressive gastrulation (dome, 30% epiboly, and 50% epiboly stages). The embryo is seen laterally, with the animal cap containing the blastula cells pointing up. Hours of development post fertilization (hpf) are indicated for each stage. Below, representative nuclear mid‐sections of nuclei of zebrafish embryos in the developmental stages are shown. (B) Distributions of counts of prominent Pol II clusters ($V\geq 0.1\;\mu m^{3}$). The distribution for the oblong stage is provided as gray area for reference in the further stages of development. Distributions are kernel‐based empirical probability densities. (C) Quantification of fluorescence intensity in whole nuclei (n=105,118,220,129,157, intensities normalized against sphere stage), data obtained from N=6,4,6,4,4 embryos from three independent experimental repeats, data points are median with 95% bootstrap confidence interval. (D) Running window analysis of nucleus Pol II Ser5P intensities (nuclei counts as above), Pol II Ser2P intensity, volume, and solidity of prominent Pol II clusters. n=994,1384,757,259,691 clusters were included in an analysis pooled over all stages. Data are shown as mean with 95% bootstrap confidence intervals. Window width 20, minimal count 12 (nucleus Pol II Ser5P) and 24 (cluster properties).

As a third and final model of differentiation, we assessed the induction of germ layers during the natural development of zebrafish embryos. We collected early zebrafish embryos during the developmental time window ranging from approximately 3.7 to 5.3 h post fertilization (hpf), thereby covering transcriptional activation of the zygotic genome (oblong and sphere stage) as well as the transition from pluripotency to persistent commitment to embryonic germ layers (dome, 30% epiboly, and 50% epiboly stages; Figure 3A) [64, 65, 66, 67, 68]. In all stages, prominent Pol II clusters could be found (Figure 3A). In the oblong and sphere stage, on average 24.9 and 22.0 prominent Pol II clusters per nucleus were detected (Figure 3B), matching previous work [69]. In the subsequent dome and 30% epiboly stages, a reduction to 4.5 and 2.5 prominent Pol II clusters, respectively, indicated a loss of most prominent clusters (Figure 3B), matching our observations in mESC differentiation and sperm precursor formation. Whole‐nucleus levels of recruited Pol II were highest in the sphere stage (Figure 3C). Levels of elongating Pol II peaked later, in the 30% epiboly stage (Figure 3C). This sequence of a transient increase in recruited Pol II followed by a transient increase in elongating Pol II corresponds to the changes in Pol II state observed for mESC and in fruit fly testes.

Similar to mESC cultures, the number of prominent Pol II clusters per nucleus in zebrafish embryos displayed a wide distribution. Therefore, we sorted the single‐cell results based on the number of clusters for further analysis (Figure 3B). Whole‐nucleus levels of recruited Pol II decreased with lower numbers of clusters, whereas levels of elongating Pol II exhibited a transient peak at ≈30 prominent clusters per nucleus (Figure 3D). The cluster volume exhibited a broader plateau, centered on ≈40 clusters per nucleus (Figure 3D). The cluster solidity exhibited an early peak at ≈55 clusters per nucleus and a second peak close to 0 (Figure 3D). Even though an analysis within each of the developmental stages could not provide continuous curves, the successive peaks in recruited Pol II and elongating Pol II (Figure 3C), the changes in number of clusters, cluster volume, and cluster solidity (Figure S8A), as well as a transient shift specifically to larger and rounder clusters of recruited Pol II (Figure S8B) could be detected. This succession of transient peaks in cluster solidity, then cluster volume, followed by elongating Pol II, and finally again cluster solidity matches the observations in mESC cultures as well as fruit fly testes, indicating a stereotypical, broadly conserved sequence of events.

### 2.4 Enhancer‐Typical Histone Acetylation Marks Decrease During Differentiation

**FIGURE 4:**
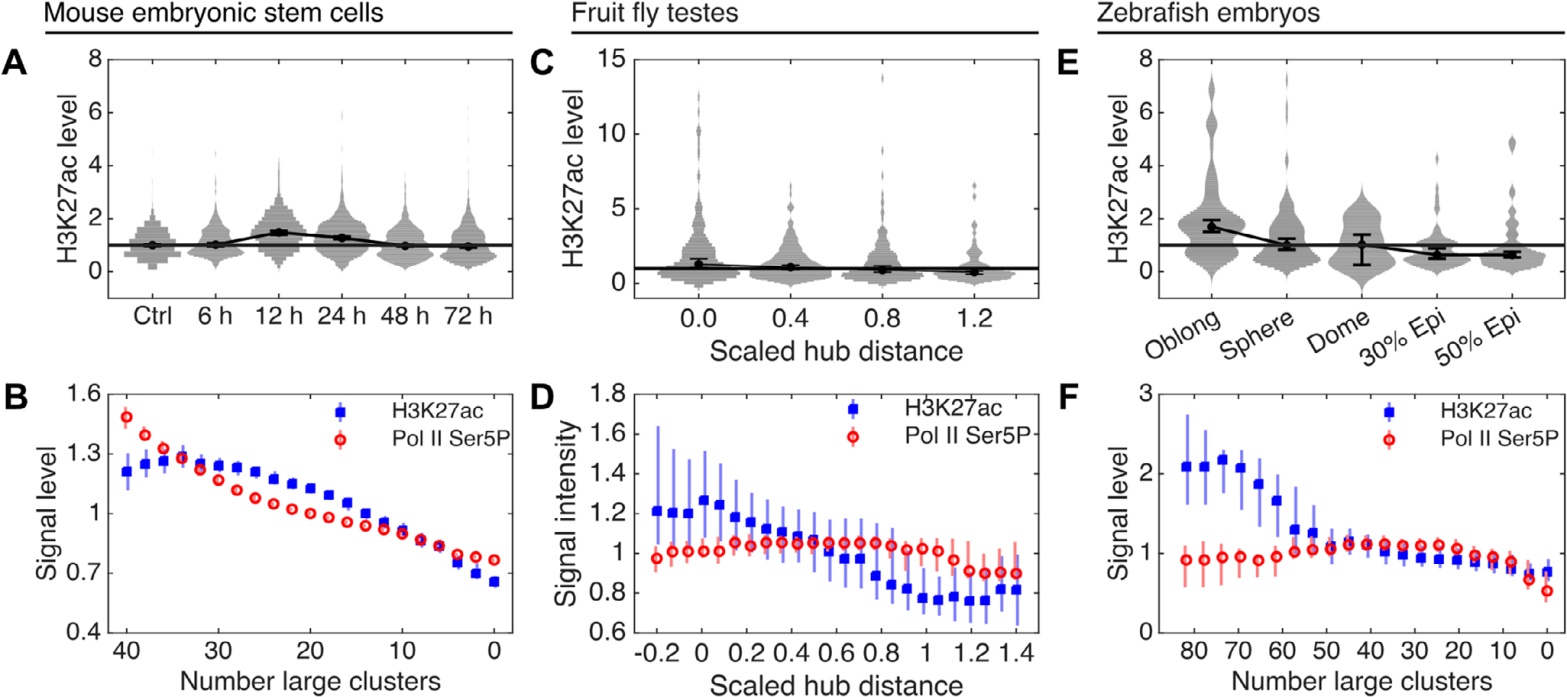
Histone acetylation indicating active enhancers transiently increases over the course of differentiation. (A) Relative H3K27ac levels in nuclei of mESCs undergoing neuronal differentiation (RHB‐A medium). Times indicate when samples were collected after beginning of the induction protocol, control (Ctrl) are uninduced mESC at 12 h after start of the induction protocol. Mean intensity values within individual nuclei were obtained by immunofluorescence and confocal microscopy. H3K27ac was normalized against nuclear volume to represent the density of the mark on chromatin, both H3K27ac and Pol II Ser5P mean intensities were normalized to the Ctrl condition as a reference. n=2087,529,464,1174,1860,2123 nuclei per condition pooled from two independent biological repeats. Individual nuclei values are shown with median and 95% bootstrap confidence interval. (B) Sliding window average for nuclei from all conditions of panel A and sorted according to number of prominent Pol II clusters in a given nucleus (8237 nuclei in total). Only windows with at least 12 nuclei were plotted, window width 10. Median values with bootstrap 95% confidence intervals. Error bars for H3K27ac and Pol II Ser5P are shifted against each other in horizontal direction for improved visibility. (C) Relative levels of H3K27ac in nuclei of cells in fruit fly testes undergoing differentiation into sperm precursor cells. Cells were binned based on distance from the apical hub (scaled hub distance, 0.4 bin width, range [−0.2,1.4]), both H3K27ac and Pol II Ser5P were normalized against the overall median of all detected nuclei. 620 nuclei were analyzed in total, pooled from 15 testes across 4 independent experimental repeats. (D) Sliding window average for nuclei based on scaled hub coordinate. Only windows with at least 12 nuclei were plotted, window width 0.55. (E) Relative levels of H3K27ac in nuclei of zebrafish embryos collected over five consecutive stages of development. Immunofluorescence, microscopy, and analysis were carried out analogous to panel A. H3K27ac and Pol II Ser5P mean intensities were normalized to the sphere‐stage level as a reference condition. n=73,127,15,76,90 nuclei were included in the analysis from 4,4,2,4,3 embryos per stage. (F) Sliding window average of H3K27ac and Pol II Ser5P signal levels for nuclei from all stages of panel E pooled (total number of nuclei 381). Only windows with at least 24 nuclei were plotted, window width 40.

Epigenetic states, including the enhancer‐associated H3K27ac mark, change rapidly during development, affecting chromatin organization and transcriptional activity of genes [70, 71, 72, 73]. High levels of the H3K27ac mark are commonly associated with active enhancers and Pol II recruitment in stem cells [74, 75]. Additionally, a partial reduction of super‐enhancer‐associated H3K27ac levels occurs with progressing differentiation in mESCs [76, 77]. In the RHB‐A medium protocol for neuronal fate induction, we found a transient increase in H3K27ac at 12 h, coinciding with the 12 h peak in Pol II phosphorylation, followed by a decay back to baseline by 48 h (Figure 4A, for Pol II phosphorylation levels see Figure 1B). The H3K27ac peak at 12 h as well as the subsequent gradual decay was confirmed by Western blotting (Figure S6). Sorting single‐cell results by number of prominent Pol II clusters per nucleus also showed a gradual loss of the H3K27ac mark in coordination with gradual reduction of recruited Pol II (Figure 4B), in line with the previously suggested interaction of H3K27ac with Pol II in mESCs.

A decrease of H3K27ac levels over the course of differentiation equally occurred in sperm cell precursor formation in fruit fly testes. Specifically, we found that H3K27ac levels decrease with progressing differentiation when assessed on the basis of geometric distance from the apical hub (Figure 4C). The decrease in H3K27ac occurred in close coordination with a transient increase in nucleus‐wide levels of recruited Pol II (Figure 4D), mirroring our results for mESC. Also in early zebrafish embryos, the H3K27ac mark has been shown to precede and support the formation of prominent Pol II clusters [17, 19, 78, 79, 80] and to be markedly reduced by the completion of gastrulation [81]. We observe that, indeed, H3K27ac levels continuously decrease over the course of germ layer induction (Figure 4E). When sorting the single‐cell results by number of prominent Pol II clusters, similar to mESC, we found that the H3K27ac mark and the level of recruited Pol II decrease in coordination (Figure 4F, for stage‐resolved Pol II phosphorylation levels, see Figure 3C), again supporting the suggested contribution of H3K27ac to the formation of prominent Pol II clusters.

### 2.5 A Surface‐Condensation Model Reproduces the Experimentally Observed Change in Shape and Dispersal of Pol II Clusters

**FIGURE 5:**
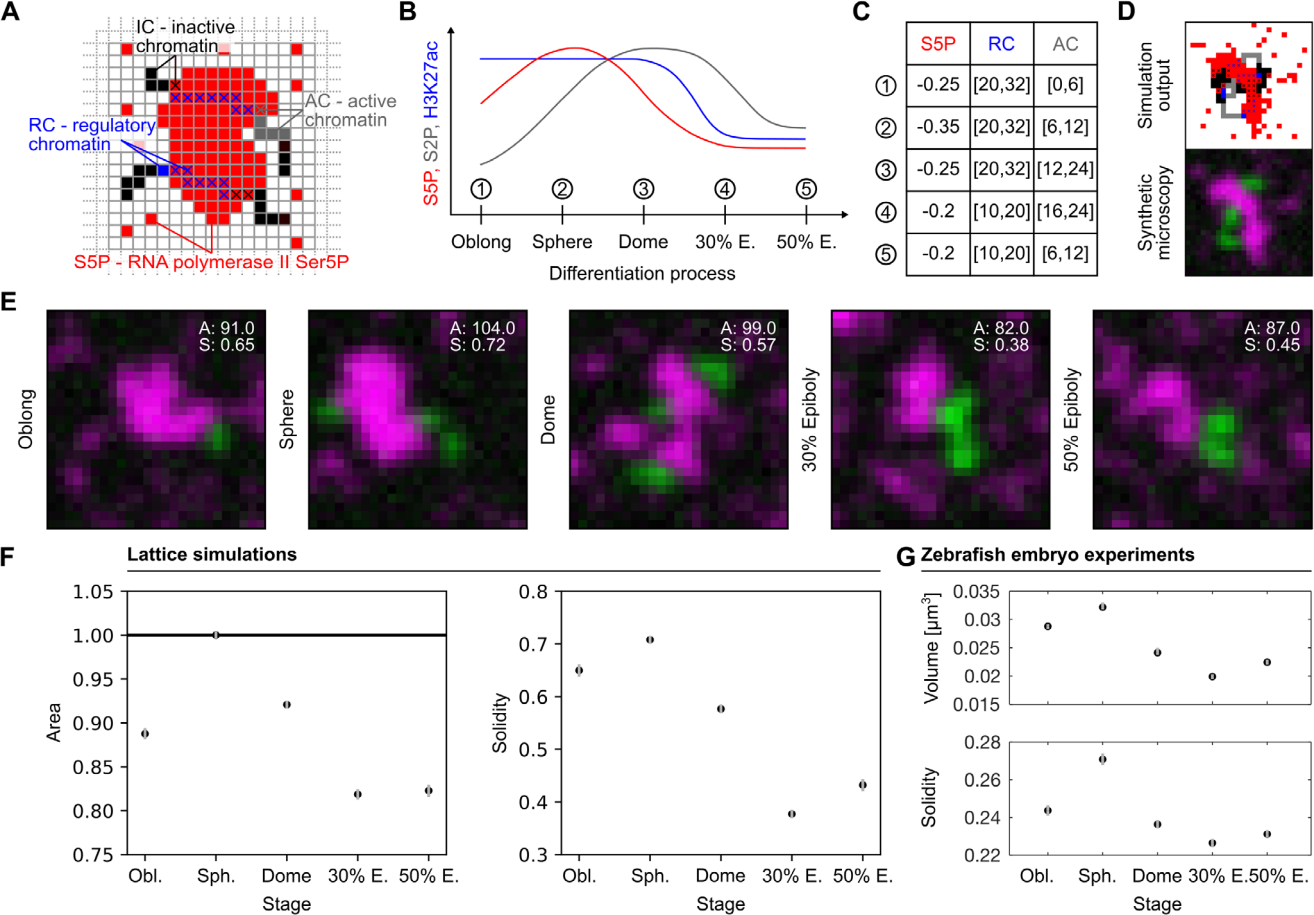
Simulations of surface condensation reproduce changes in the cluster area and solidity observed throughout the differentiation process. (A) Lattice representation of the surface‐condensation model, consisting of polymers with three subregions (IC, inactive chromatin; RC, regulatory chromatin; AC, active chromatin) and liquid material that can form clusters and contains recruited RNA Pol II (S5P). (B) Sketch of experimental results (zebrafish embryos) that summarizes the changes in levels of recruited Pol II (S5P), transcription activity (S2P) and available surface (H3K27ac). (C) Selection windows of simulation parameters representing the different developmental stages. (D) Representative simulation output and according synthetic microscopy images. Magenta represents S5P (red) and green S2P (gray) within the blurred image, matching the color scheme used for the display of experimental data. (E) Synthetic microscopy example images for each stage, area (A) and solidity (S) as indicated. (F) Cluster area and solidity in synthetic microscopy data from lattice simulations in the selection windows defined in panel C. Data are mean with 95% confidence bootstrap confidence interval. (G) Cluster volume and solidity in experimental data for indicated stages of zebrafish embryo development. To allow comprehensive detection of volume changes, a lowered minimal volume threshold of $0.01\;\mu m^{3}$ was used. Nuclei count: 129, 157, 220, 105, 118; cluster count: 11496, 12151, 10562, 7269, 11157, shown as mean with 95% bootstrap confidence interval.

Considering the similarity of the observations in all three experimental models of differentiation, we theorized that cluster formation and dispersal proceed on the basis of a general underlying biophysical mechanism. Accordingly, we aimed to provide a generalized theoretical model for the formation of transcriptional clusters. Previous work proposed a surface‐condensation model of transcriptional cluster formation, which can accept H3K27ac, recruited Pol II, and elongating Pol II levels as input parameters [19]. In particular, H3K27ac‐marked super‐enhancers facilitate the condensation of a liquid phase that contains recruited Pol II, whereas increased levels of elongating Pol II induce the unfolding or even dispersal of transcriptional condensates formed in this fashion (Figure 5A). These aspects of the model imply that the reduction in super‐enhancers that are available as condensation surfaces as well as increased transcription levels seen in our experiments could reduce the overall number of prominent transcriptional clusters.

For numerical simulations of the theoretical model, we adapted the coarse‐grained lattice kinetic Monte‐Carlo (LKMC) approach [82] from previous work on surface condensation (Figure 5A) [19]. With a proper choice of parameters, this model can describe the formation of surface‐supported condensates in the sub‐saturated regime, where bulk phase separation does not occur. The model relies on generic interaction laws, and thus delivers a first‐principle theoretical prediction of the overall system dynamics. The lattice simulations contain single particles that represent the material forming the clusters enriched in recruited Pol II, which exhibit self‐affinity (S5P, red, interaction energy $w_{S5P-S5P}<0$). Chromatin is represented by block copolymer chains with different subregions: regions of “inactive” chromatin (IC, black, self affinity $w_{IC-IC}<0$), followed by regions of regulatory chromatin (RC, blue) that have an affinity for the red species ($w_{RC-S5P}<0$) and thereby can serve as a condensation surface, and directly adjacent regions of active chromatin harboring elongating Pol II (AC, gray) that is repelled by red particles ($w_{AC-S5P}>0$). We performed simulations containing polymer chains with randomized lengths of blue and gray block regions for different affinities between red particles, which all fall within the sub‐saturated regime with respect to the red particles [19], thereby obtaining a total of 300 different lattice simulation runs, from which simulations could be selected that fall within specific parameter windows (for more details, see Methods Section).

To test our model, we assessed whether changes in cluster shape match the observations in zebrafish embryos, where stages can be clearly separated experimentally and compared against simulation results. We used the experimentally determined, nucleus‐wide levels of available condensation surface (H3K27ac), recruited Pol II, and transcriptional activity (Figure 5B). In particular, we considered initially elevated and subsequently decreasing levels of H3K27ac, as observed in all experimental model systems (Figure 4). A subsequent transient peak in Pol II Ser5P was clear from stage‐resolved assessment in mESC (Figure 1B and Figure S4B) and zebrafish embryos (Figure 3C) and variably apparent in fruit fly sperm precursor formation (Figures 2C and 4D). A further delayed, transient peak in transcriptional activity in the vicinity of large clusters of recruited Pol II could be seen for the mESC differentiation (Figure 1D and Figure S4C), sperm precursor formation (Figure 2C), and zebrafish embryos (Figure 3D). For the concrete comparison against experimental data, we select those simulations that represent the five developmental stages of zebrafish embryos, which we see among our model systems as the system with the clearest staging of differentiation. To this end, we defined corresponding selection windows that can be applied to the randomly assigned polymer assemblies (see Figure 5C). To compare simulation outputs to the microscopy data, we produced synthetic microscopy images by adding a Gaussian blur filter approximating limited microscope resolution and detector noise (Poisson noise) to the lattice model output (see Figure 5D). To supplement our quantitative analysis, we also provide representative synthetic microscopy images for each stage, which compare favorably with clusters seen in actual microscopy data (Figure 5E). We then extracted cluster area and solidity with an image analysis pipeline that corresponds to the analysis of our actual microscopy images (Figure 5F). The area as well as the solidity of clusters transiently increased at the sphere stage, and dropped to the lowest values at the 30% epiboly and 50% epiboly stages (Figure 5F). These observations match the results obtained for Pol II cluster volume and solidity in the experimental data (Figure 5G). Taken together, simulations based on condensation facilitated by super‐enhancers as a condensation surface reproduced key features of the change in shape and dispersal of transcriptional clusters as observed in our experiments.

### 2.6 Chemical Perturbations Confirm Super‐Enhancers Acting as Condensation Surfaces and Transcribed Genes as Dispersing Amphiphiles

**FIGURE 6:**
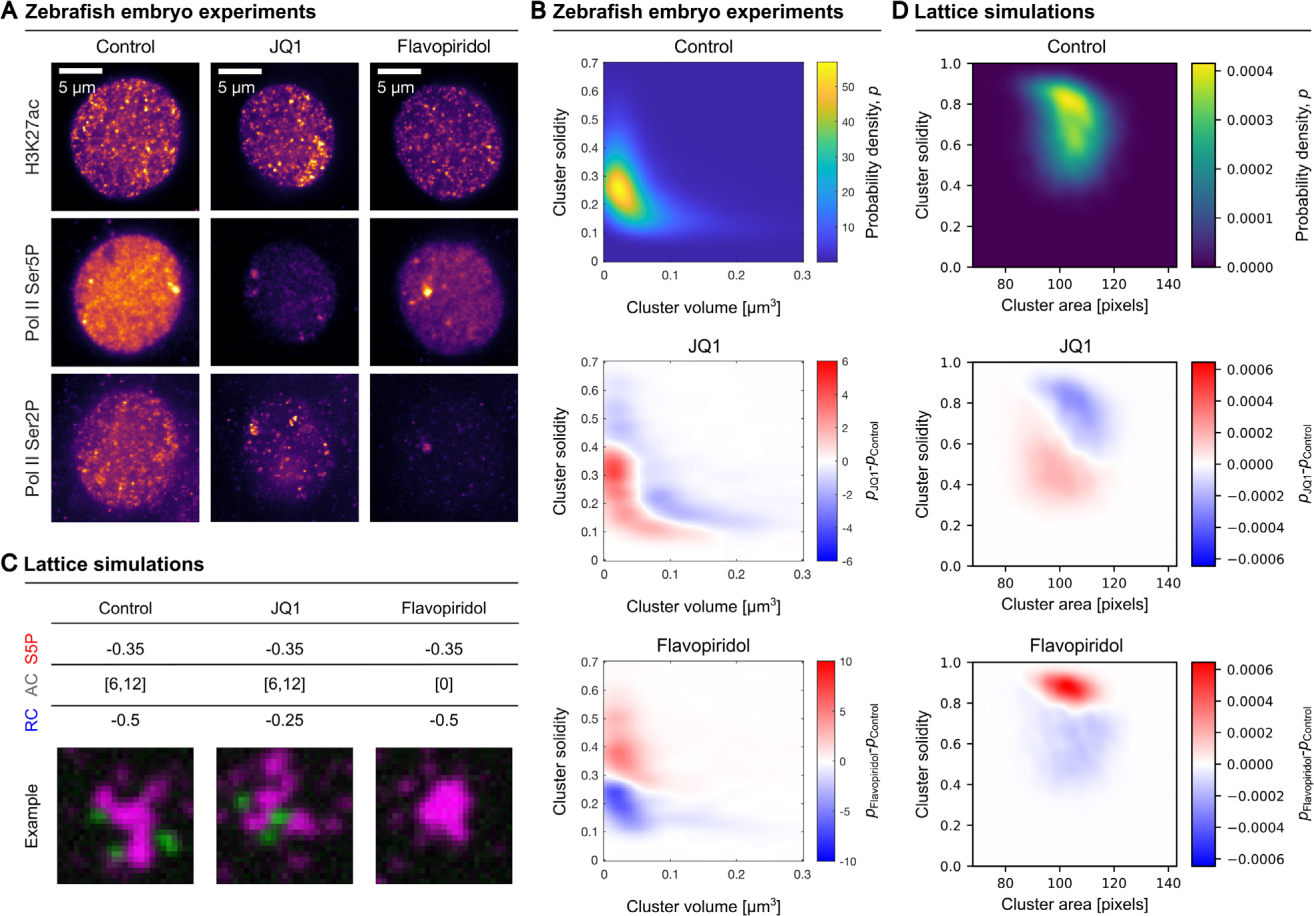
Acute inhibitor treatments test the roles of super‐enhancers as condensation surfaces and transcribed genes as dispersing amphiphiles. (A) Example micrographs of nuclear mid‐sections; H3K27ac, Pol II Ser5P, and Pol II Ser2P labeled by immunofluorescence. (B) Two‐dimensional distributions of clusters in volume‐solidity space, assessed via kernel‐based probability density estimation. For the control condition, probability density p is shown. For inhibitor‐treated conditions, probability density differences are shown to display shifts in the cluster distribution. (C) Parameter changes and example images for simulated treatments. (D) Two‐dimensional distributions of clusters in area‐solidity space for lattice simulations. n=50 simulations were included in the analyses for each condition.

Our findings suggest that a reduction in the levels of H3K27ac‐marked super‐enhancers and an increase in transcriptional activity can explain the loss of prominent transcriptional clusters during differentiation. To test both parameters as causal, we applied the respective chemical inhibitors JQ1 and flavopiridol to zebrafish embryos and collected embryos for imaging at the sphere stage. Upon treatment with the BET domain inhibitor JQ1 for 30 min, a marked reduction of nucleus‐wide levels of recruited and elongating Pol II confirmed inhibitor efficacy (Figure 6A). The distribution of Pol II clusters shifted toward lower cluster volume and solidity, confirming that JQ1‐induced perturbation of super‐enhancers compromises the integrity of prominent transcriptional clusters (Figure 6B). Upon treatment with the CDK9 inhibitor flavopiridol for 30 min, as expected, signal of elongating Pol II was largely abolished (Figure 6A). Relevant levels of recruited Pol II were retained, but Pol II clusters appeared to have changed shape (Figure 6A). Indeed, a systematic assessment revealed that the shape of clusters was shifted toward higher solidity across the entire range of cluster volumes, indicating an overall rounding‐up of Pol II clusters (Figure 6B). To mimic the effect of JQ1 in the lattice simulations, we lowered the affinity between Pol II and regulatory chromatin in the lattice simulations (Figure 6C). As a result, a shift to lower cluster volume and solidity occurred that closely matched the experimentally observed shift (Figure 6D). To mimic the effect of flavopiridol, we excluded gray regions representing transcriptional elongation (Figure 6C). A shift toward higher solidity occurred that, again, closely matched the experimentally observed shift (Figure 6D). We conclude that super‐enhancer status as well as transcriptional activity causally contribute to the shape of transcriptional clusters, in line with their respective roles as condensation surfaces and drivers of unfolding in the surface‐condensation model.

## 3 Discussion

**FIGURE 7:**
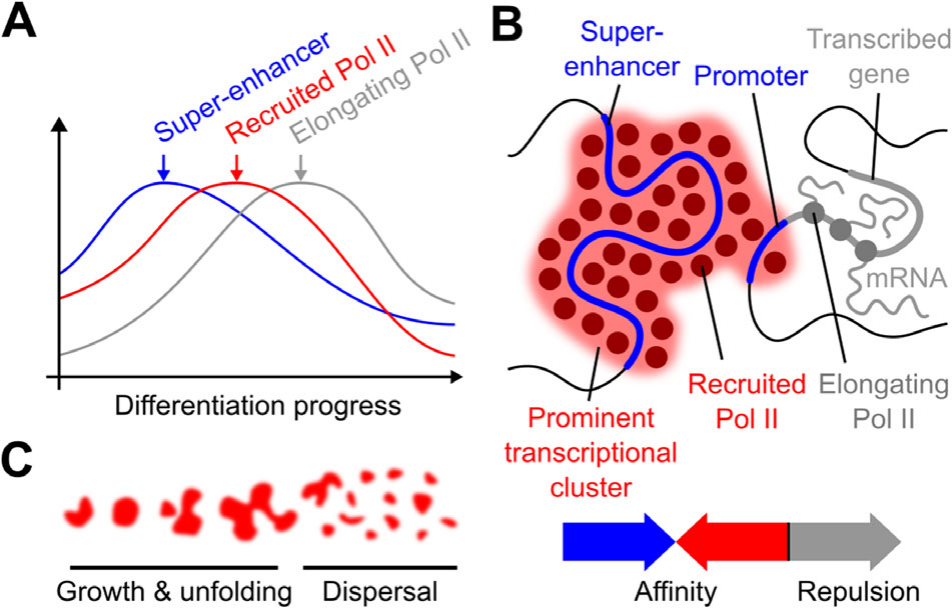
Stem cell‐specific transcriptional condensates follow a trajectory of change in shape and subsequent dispersal in line with a surface‐condensation model. (A) Over the course of differentiation, subsequent peaks occur for active enhancer marks, global levels of recruited Pol II, and levels of elongating Pol II in proximity to prominent transcriptional clusters. (B) Formation of prominent transcriptional clusters is explained by a model where transcriptional activators (red, including recruited Pol II) condense on regulatory chromatin regions, most prominently super‐enhancers (blue). These clusters unfold and partially disperse due to the exclusion of transcribed genes (gray) from the condensate material. (C) These surface‐condensation mechanisms, over the course of differentiation, result in growth, unfolding, and ultimately dispersal of prominent transcriptional condensates.

In this study, we assessed how stem cell‐specific, prominent Pol II clusters change shape and, ultimately, disperse over the course of differentiation. We found a sequence of coordinated changes in global transcription control state and the shape of clusters, which was conserved across different experimental models of differentiation. At its completion, this sequence resulted in a configuration of mostly smaller Pol II clusters, which is typical of differentiated cells. In particular, levels of active regulatory elements, levels of recruited Pol II, and levels of elongating Pol II exhibited successive transient peaks over the course of differentiation, in respective order (Figure 7A). Our polymer simulations indicated that these regulatory changes result in a stereotypical trajectory through the space of possible configurations of transcriptional condensates formed at regulatory genomic elements (Figure 7B). In these simulations, the availability and length of regulatory genomic regions, which act as condensation surfaces, controlled the occurrence of large clusters (marked via recruited Pol II, Figure 7B). The global level of recruited Pol II additionally modulated the size of these clusters, whereas elongating Pol II in proximity to these clusters resulted in their unfolding and dispersal (Figure 7B). Our findings explain cluster formation, change in shape, and dispersal as a trajectory fully in line with a surface‐condensation model, which plays out in a regime below the saturation concentration needed for canonical liquid–liquid phase separation (Figure 7C). The combination of conservation across experimental systems and a coherent biophysical mechanism supports the surface‐condensation model as an explanation of how prominent transcriptional clusters are formed in stem cells and, with progressing cell fate choice, are converted into smaller clusters that are typical of differentiated cells [1, 25, 26].

The involvement of liquid‐phase properties in the formation of transcriptional clusters is an active area of discussion [23, 24, 47, 50]. Several studies have discussed a role for liquid–liquid phase separation in the formation of Pol II clusters, but also revealed that in vivo concentrations typically fall below the saturation concentration required for the formation of phase‐separated droplets [12, 27, 28, 29, 30, 31, 32, 33]. Surface condensation mediated by DNA strands with transcription factor binding motifs provides a consistent mechanism for the formation of condensates at sub‐saturated concentrations [38, 39]. Here, the number and spatial proximity of relevant genomic binding sites controls the formation, size, and location of transcription factor clusters, acting in line with a surface‐condensation model [83]. A similar contribution to condensate formation by super‐enhancer regions was previously observed [27, 30]. Further, the formation of Pol II clusters in zebrafish embryos could be reproduced by simulations of surface condensation, where regulatory chromatin acts as a condensation surface [19], as well as a synthetic system, where DNA‐nanostructures condense on DNA strands [40]. These works support a conceptualization of stem cell‐specific, prominent transcriptional clusters as surface condensates formed on regulatory chromatin—the first main mechanism of our proposed surface‐condensation model.

Our observations of changes in cluster shape relate to previous findings that, once formed, transcriptional clusters exhibit a complex internal organization, including compartments at the scale of ∼100 nm that correspond to consecutive steps of transcription regulation [9, 55, 56, 84, 85]. The spatial separation of Pol II recruitment and transcript elongation, for example, could be explained in terms of the exclusion of transcribed genes and their RNA transcripts from a liquid phase enriched in recruited Pol II [57, 86]. Here, transcribed genes take on the role of an effective amphiphile, which can unfold or even split apart transcriptional clusters [16, 19, 20]. A comparable dispersal of DNA‐nanomotif condensates by synthetic amphiphile particles has been demonstrated in a fully artificial, cell‐free model system [58]. Together with the dispersing effect of transcribed genes in our simulations, these findings emphasize that RNA transcripts emerging during ongoing transcription induce an incompatibility of the transcribed gene with transcriptional condensates. This incompatibility, in turn, results in the exclusion of transcribed genes from transcription control condensates, accompanied by dispersal of the condensate. This dispersal via an amphiphilic effect of transcribed genes represents the second main mechanism of our surface‐condensation model.

Although our work indicates a conserved biophysical mechanism for the formation and dispersal of stem cell‐specific transcriptional clusters, the question remains how such clusters might contribute to the particular transcriptional programs associated with stem cells and their differentiation [1, 87, 88]. Work over the recent years has contributed to a model of gene‐regulatory hubs, where multiple enhancers come into contact and provide a localized context that can be visited by genes, which thereby undergo transcriptional regulation [89, 90, 91, 92, 93]. These hubs sequester CDK9, requiring genes to engage with the hubs for their activation [94]. In embryonic development, persistent enhancer hubs are, indeed, visited by different genes depending on differentiation and transcriptional activation [95, 96]. Corresponding transient interactions of genes with enhancers that control their transcription were seen in live imaging [97, 98] as well as pseudo‐time reconstruction from fixed cells [20]. Hubs involving enhancers and genes also form prior to transcriptional activation, with an involvement of recruited Pol II, seemingly providing a configuration that is poised for subsequent transcriptional activation during differentiation processes [45, 99, 100]. In line with this idea of preparation prior to transcriptional activation, Pol II recruitment increases [101, 102] and gene–gene and gene–enhancer loops [99, 103] form prior to developmental transcriptional activation. Additionally, super‐enhancers underlying long‐range contacts (≈1 Megabase and above) as well as gene expression changes are partially deactivated upon exit from pluripotency and activated upon induction of new cell types, supporting a model where transcriptional hubs would be formed and subsequently dispersed via the activation and deactivation of super‐enhancers [76, 77, 89, 104]. In mice, specific enhancer‐promoter interactions increase with differentiation into different tissue types [105]. Also in mice, neural differentiation leads to changes specifically in promoter‐enhancer contacts in vitro [104] and the organization of long genes [106]. In zebrafish embryos, super‐enhancers become increasingly compacted [107] and contribute to long‐range contacts [108] during early development. In fruit flies, long‐range contacts are equally established prior to and independently of transcriptional activation, via clustering of the pluripotency‐associated transcription factor Zelda [109, 110, 111]. Similar clustering of pluripotency‐associated transcription factors and chromatin modifiers into “transcription bodies” was seen also in zebrafish [18, 21] as well as in mESCs [12]. In human cells, local accumulations of transcription factor binding sites occur in regions where cohesin accumulates, pointing toward possible transcription factor clustering [112]. In conclusion, the spatial clustering of enhancers and transcription‐associated factors into hubs emerges as a potentially general mechanism by which stem cells prepare for subsequent differentiation into specific cell types. Our work provides a biophysically consistent surface‐condensation mechanism to dynamically form and subsequently disperse these transcriptional hubs, enabling their seemingly central role in cell fate choice.

## 4 Methods Section

### 4.1 Mouse Embryonic Stem Cell Culture

#### 4.1.1 Cell Culture and Differentiation

The Mouse ESC 46C cell line (male origin, passage N + 23, derived from E14tg2a and expressing GFP under Sox1 promoter; [113]) were a gift from Prof. Domingos Henrique (Instituto de Medicina Molecular, Faculdade Medicina Lisboa, Lisbon, Portugal). mESCs (ESC46C) were grown as described in [45]. In brief, mESCs were grown at 37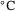 in a 5% (v/v) ${\mathrm{CO}}_{2}$ incubator in GMEM medium (Invitrogen), supplemented with 10% (v/v) Fetal Calf Serum (FCS; BioScience LifeSciences), 2000 U/mL LIF (Millipore), 0.1 mM β‐mercaptoethanol (Invitrogen), 2 mM L‐glutamine (Invitrogen), 1 mM sodium pyruvate (Invitrogen), 1% penicillin–streptomycin (Invitrogen), 1% MEM non‐essential amino acids (Invitrogen) on gelatin‐coated (0.1% v/v) Nunc T25 flasks. The medium was changed every day, and cells were split every other day. Before sample collection (control time point), mESCs were plated on gelatin‐coated (0.1% v/v) Nunc 10‐cm dishes in serum‐free ESGRO Complete Clonal Grade Medium (Millipore) to which was added 1000 U/mL LIF. Medium was changed to the cells every 24 h and cells were collected at 48 h. mESCs were routinely tested for mycoplasma contamination using the MycoSPY Mycoplasma detection kit (Biontex, # M020‐050) according to the manufacturer's instructions.

Early neuronal differentiation was carried out as described in [45]. mESCs were plated with high density (1.5 × 10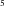 cells/${\mathrm{cm}}^{2}$) in serum‐free ESGRO Complete Clonal Grade Medium (Millipore) to which 1,000 U/mL LIF was added. After 24 h, mESCs were washed 3 times with PBS (without magnesium and calcium), incubated in PBS for 3 min at room temperature, and then dissociated by incubating in 0.05% (v/v) Trypsin (Gibco) for 2 min at 37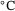. mESCs were plated onto 0.1% (v/v) gelatin‐coated 10‐cm dishes (Nunc) at 1.6$\times 10^{6}$ cells/dish in RHB‐A (Takara–Clontech), changing media every 24 h. These differentiating cells were collected after 3, 6, 12, 24, and 48 h in RHB‐A. Alternatively, for the LIF withdrawal experiment, mESCs were plated onto 0.1% (v/v) gelatin‐coated 10‐cm dishes (Nunc) at 1.6$\times 10^{6}$ cells/dish in GMEM medium (see above) without LIF. Differentiating cells were collected after 3, 6, 12, 24, and 48 h of LIF withdrawal. Upon harvesting, cell suspension was fixed with 2% PFA for 30 min at room temperature and then centrifuged (5 min, 320 g). To increase the mechanical stability of cells, the supernatant was removed and a secondary fixation step was carried out by applying 8% formaldehyde in PBS for 30 min at room temperature, followed by centrifugation (5 min, 320 g) and removal of the supernatant.

### 4.2 mRNA Expression

RNA was isolated to test the expression of differentiation markers by quantitative PCR (qPCR) as previously described [45]. Total RNA was extracted using TRIzol (Invitrogen, # 15596026) according to the manufacturer's instructions. TRIzol samples were incubated in chloroform (1:0.2 sample:chloroform ratio) for 3 min at room temperature. After centrifugation (16,260g, 15 min, 4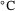), the upper aqueous phase was transferred to a new tube and the RNA was precipitated using High Performance Liquid Chromatography (HPLC)‐grade isopropanol. After centrifugation (16,260×g, 10 min, 4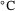), the RNA pellet was washed twice with 75% ethanol and eluted in RNAse‐free water. The DNA was removed with TURBO DNAse (Thermo Fisher, # AM2238) according to the manufacturer's instructions, and 1μg of the purified RNA was reverse transcribed with 50 ng random primers and 10 U SuperScript II Reverse Transcriptase (Invitrogen, # 18064‐071) according to the manufacturer's instructions. The synthesized cDNA was diluted 1:10, and 2.5 μL used for qRT‐PCR with the 2 SensiMix SYBR No‐ROX (Thermo Fisher, # AB1159A) according to the manufacturer's instructions, and primers that span exon–exon junctions. The amplification products were normalized to β‐actin (Actb) as a housekeeping gene (primers see Table 1).

**TABLE 1:** Primer pairs used for qPCR amplification.

Target	Direction	Sequence
β‐actin	Fw	TCTTTGCAGCTCCTTCGTTG
	Rev	ACGATGGAGGGGAATACAGC
Nanog	Fw	ATGAAGTGCAAGCGGTGGCAGAAA
	Rev	CCTGGTGGAGTCACAGAGTAGTTC
Oct4	Fw	CTGAGGGCCAGGCAGGAGCACGAG
	Rev	CTGTAGGGAGGGCTTCGGGCACTT
Blbp	Fw	GGGTAAGACCCGAGTTCCTC
	Rev	ATCACCACTTTGCCACCTTC
Hes5	Fw	AAGTACCGTGGCGGTGGAGAT
	Rev	CGCTGGAAGTGGTAAAGCAGC

#### 4.2.1 Protein Extraction and Western Blotting

Whole cells were washed once with room temperature 1x PBS (Bio&Sell, # BS.L182‐50) and lysed in RIPA buffer consisting of 1x PBS pH 7.4, 0.5% sodium deoxycholate (Sigma–Aldrich, # D6750‐100G), 1% IGEPAL CA‐630 (Sigma–Aldrich, # I8896‐100ML), 1x complete protease inhibitor cocktail (Roche, # 11697498001), 1 mM PMSF (Sigma–Aldrich, # 93482‐50ML‐F), and 1 mM sodium orthovanadate (BioLabs, # P0758S). Lysates were incubated on ice for 30 min and cleared by centrifugation for 30 min at 14,000×g, after which the supernatant was transferred to a fresh tube. Protein concentration was determined using the DC Protein Assay Kit (Bio‐Rad, # 5000112), and a Tecan Infinite 200 Pro plate reader (Tecan Austria GmbH, REF 30050303) according to the manufacturer's instructions. For Western blotting, approximately 20 μg total protein per sample were diluted in RIPA buffer and mixed with 1x final concentration of Laemmli loading buffer, (Bio‐Rad, # 161‐0747), (loading buffer was supplied with 10% of β‐mercaptoethanol, Sigma–Aldrich, # M6250‐100ML). Samples were denatured for 5 min at 95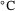 and loaded onto 4%–12% gradient mPAGE Bis‐Tris gels (Millipore, # MP41G15). Electrophoresis was performed using Tris‐MOPS‐SDS running buffer (GenScript, # M00138). PageRuler Plus Prestained Protein Ladder (Thermo Scientific, # 26619), was used as molecular weight marker. Proteins were transferred using the Trans‐Blot Turbo RTA Mini 0.2 μm Nitrocellulose Transfer Kit (BioRad, #1704270) and the semi‐dry Trans‐Blot Turbo System (Bio‐Rad, # 1704150), for 30 min with the standard protocol. Transfer efficiency was verified by Ponceau S staining (0.1% Ponceau S (Roth, # 59382), 1% acetic acid (TH. GEYER, # 2234.1000) in distilled water). Membranes were washed twice for 5 min in 1x TBST (500mM NaCl (Roth, # 3957.2); 3.5 mM Tris (Roth, # AE15.2); 1.65 mM Tris‐HCl (Roth, # 9090.3) in distilled water) supplemented with 0.1% Tween‐20 (Sigma–Aldrich, # P1379‐500ML). After washing, membranes were blocked for 1 h at room temperature in blocking buffer consisting of 5% low‐fat blotting‐grade milk powder (Roth, # T145.3) in 1x TBST. Following incubation with primary and secondary antibodies (see Table 2), membranes were washed three times in 1x TBST. Membranes were treated with Western Lightning Plus‐ECL substrate (PerkinElmer, # NEL104001EA) following the manufacturer's recommendations and western blotting images were acquired using a Fusion FX7 chemiluminescence detection system (Peqlab).

**TABLE 2:** List of primary and secondary antibodies used for Western blots.

Target	Type	Clone/Conjugate	Supplier	Cat. No.
Primary antibodies				
β‐Tubulin	Rabbit IgG		Abclonal	AC015
GAPDH	Mouse IgG		Cell signaling	97166S
Total RNAPII	Rabbit IgG		Cell signaling	14958S
H3K27ac	Rabbit IgG		Active Motif	39134
RNAPII‐S5P	Mouse IgG	4H8	BioLegend	904001
Secondary antibodies				
Mouse IgG	Anti‐mouse IgG (Goat)	HRP	Abcam	ab6789
Rabbit IgG	Anti‐rabbit IgG (Goat)	HRP	Dako	P0448

#### 4.2.2 Immunofluorescence

For all steps that include replacement of liquids, sample tubes were centrifuged (1 min, 800g) before liquid removal. To begin immunostaining, cells were fixed again with a higher concentration of formaldehyde (8% FA in PBST, 20 min, room temperature), then washed three times with PBST. Cells were permeabilized with 0.5% Triton X‐100 in PBS (10 min, room temperature), then washed three times with PBST. Cells were blocked with 4% BSA in PBST (30 min, room temperature). Cells were incubated in primary antibody mix (1:1,000 rabbit anti‐Pol II Ser2P, 1:1,000 rat anti‐Pol II Ser5P in 4% BSA in PBST, Table 3) overnight at $4^{\circ }C$, then washed three times with PBST. Cells were incubated in secondary antibody mix (1:1,000 anti‐rat IgG Alexa 594 and 1:1,000 anti‐rabbit IgG Alexa 488 in 4% BSA in PBST, Table 3) overnight at 4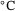, then washed three times with PBST. In mESC samples where, additionally, we detected H3K27ac by immunofluorescence (Figure 4A,B), labeling was carried out in two rounds to prevent cross‐detection of rat primary antibodies by anti‐mouse secondary antibodies. Specifically, we first applied mouse anti‐Pol II Ser2P primary antibodies and anti‐mouse IgM secondary antibodies, followed by rat anti‐Pol II Ser5P and rabbit anti‐H3K27ac primary antibodies and anti‐rabbit and anti‐rabbit secondary antibodies (see Table 3, all 1:1,000 in 4% BSA in PBST). Samples were post‐fixed with 8% formaldehyde in PBST for 15 min at room temperature, then washed three times with PBST. For sample mounting, liquid was removed as far as possible without removing cells, and replaced by VectaShield H‐1000 with 2 μM Hoechst 33342 (1:10,000 dilution from 20 mM stock). Cells were resuspended and transferred to 8‐chamber microscopy slides (ibidi μ‐slide, glass bottom #1.5 selected cover glass) using a P200 micropipette (100 μL per chamber).

**TABLE 3:** List of primary and secondary antibodies used for immunofluorescence.

Target	Type	Clone/Conjugate	Supplier	Cat. No.
**Primary antibodies**				
Pol II S5P	Mouse IgG	4H8	Abcam	ab5408
Pol II S5P	Rat IgG	3E8	Active motif	61986
Pol II S2P	Mouse IgM	H5	Biolegend	920204
Pol II S2P	Rabbit IgG	EPR18855	Abcam	ab193468
H3K27ac	Rabbit IgG	EP16602	Abcam	ab177178
**Secondary antibodies**				
Anti‐mouse IgM	Goat	Alexa 594	Invitrogen	A21044
Anti‐rabbit	Donkey	Alexa 488	Invitrogen	A21206
Anti‐rabbit	Goat	Alexa 647	Thermofisher	A21244
Anti‐rabbit	Goat	Alexa 488	Thermofisher	A11006
Anti‐rat	Goat	Alexa 594	Invitrogen	A11007
Anti‐rat	Goat	Alexa 488	Thermofisher	A11066
Anti‐rat	Goat	Alexa 647	Thermofisher	A21247

All primary antibodies were validated in a previous publication [19].

For the scoring of mitotic cells, the immunofluorescence protocol was carried out up until before treatment with 0.5% Triton X‐100 in PBS. Instead, PBST was removed from the tube containing the pelleted cells. Cells were resuspended in Vectashield H‐1000 with 2 μM Hoechst 33342 and transferred for imaging in 8‐chamber microscopy slides.

#### 4.2.3 Microscopy

The prepared samples were recorded using a VisiTech iSIM high‐speed super‐resolution confocal microscope based on the instant‐SIM principle [59], built on a Nikon Ti2‐E stand. A Nikon 100× oil immersion objective (NA 1.49, SR HO Apo TIRF 100xAC Oil) and excitation lasers at 405, 488, 561, and 640 nm were used. Two Hamamatsu ORCA Flash4.0 V3 cameras were used for dual color acquisition on the basis of a 561‐nm long‐pass beam splitter. A lateral resolution of ≈125nm and an axial resolution of ≈350nm can be achieved. A sphere with the minimal cluster volume $0.1\;\mu m^{3}$ used in our analysis would have a diameter of 576nm, providing a lower bound estimate that indicates that the internal structure of large clusters can, indeed, be assessed with this optical resolution. In a given experimental repeat, the illumination and acquisition setting were kept constant. For the scoring of mitotic cells, a 40× water immersion objective was used (Apo LWD 40× WI S DIC N2, NA 1.15). Images of mESC underlying Figure 4A,B were recorded after the iSIM system was upgraded by a replacement of the dual acquisition cameras by two Hamamatsu ORCA‐Quest cameras.

#### 4.2.4 Image Analysis

Depending on the data set, nuclei were segmented using the Pol II Ser5P channel (Figure 1 and Figure 4) or the Hoechst channel (Figure 4A,B), applying a Gaussian blur (kernel width σ=1.0μm) for the foreground and for background subtraction (σ=10μm). Nuclei masks were segmented using Otsu threshold binarization, followed by a 3D hole‐filling topological operation. To exclude edge effects, nuclei masks were eroded with a disk of diameter 0.5μm. Only nuclei of minimal volume of $80\;\mu m^{3}$ and minimal solidity 0.8 were retained for further analysis. For cluster detection, Gaussian blur was applied for segmentation of the Pol II Ser5P channel (σ=0.06μm), with background removal (Gaussian blur with σ=0.1μm). Segmentation of Pol II Ser5P objects was done with the robust background threshold method (2 standard deviations above mean of all pixel intensities). Objects below a minimal volume of $0.005\;\mu m^{3}$ were removed from further analysis. Individual objects were then joined into clusters using DBSCAN with ε=0.5μm. Fluorescence intensities inside clusters were mean intensities averaged over all pixels in the cluster segmentation mask and normalized against the median intensities over the entire nucleus that a given cluster resides in.

For the quantification of the number of prominent clusters and level of the H3K27ac chromatin mark, corrections had to be applied to transition from measured values to physically appropriate values. The directly measured number of large clusters in a given nucleus, ${\tilde{n}}_{clusters}$, can be affected by parts of nuclei cut off close the edges of the microscopy z‐stack. The corrected number was calculated as

(1) $$\begin{array}{c} n_{clusters}={\tilde{n}}_{clusters}\frac{\mathrm{median}(V)}{\tilde{V}}={\tilde{n}}_{clusters}\frac{\pi d^{3}}{6\tilde{V}}, \end{array}$$

using the median nuclear volume in a given condition, median(V), to scale the number of clusters and thereby compensate for the missing parts of nuclei at stack edges. The same correction for the number of clusters was used in the analysis of all experimentally obtained microscopy data in this study. The chromatin mark H3K27ac was measured as mean intensity inside segmented nuclei, ${\tilde{I}}_{K27ac}$, and needed to be converted to represent a normalized level of H3K27ac per chromatin,

(2) $$\begin{array}{c} l_{K27ac}=\frac{I_{K27ac}}{V}\langle \frac{V^{ref}}{I_{K27ac}^{ref}}\rangle =\frac{I_{K27ac}}{d^{3}}\langle \frac{{\left(d^{ref}\right)}^{3}}{I_{K27ac}^{ref}}\rangle , \end{array}$$

where the index ref indicates measurements from a reference condition and ⟨⋯⟩ indicates the arithmetic mean over all nuclei of that reference condition. To inherently overcome z‐stack edge effects, here the proportionality $V=\pi /6\times d^{3}$ was used to replace volumes V by object diameters d measured in the xy‐plane, assuming approximately spherical nuclei. The diameter was calculated as

(3) $$\begin{array}{c} d=\sqrt{d_{x}\cdot d_{y}}, \end{array}$$

where the measured horizontal ($d_{x}$) and vertical ($d_{y}$) extent of the object bounding box are combined to estimate the value of d. Note that the bounding box captures the largest extent across all z‐sections, inherently capturing the z‐position of the widest extent of a theoretically assumed spherical object. The same correction was used for all quantifications of H3K27ac levels from experimentally obtained microscopy data in this study.

For the scoring of mitotic cells, nuclei and mitotic chromosomes were segmented as described above, with the adjusted parameters: foreground smoothing kernel width σ=0.5μm, background smoothing kernel for background subtraction σ=6μm, minimal object volume $1.0\;\mu m^{3}$, minimal object solidity 0.1. For each object, the mean and the coefficient of variation (CoV, mean divided by standard deviation) of the DNA pixel intensities were calculated. Within each condition, a robust background threshold (mean+1.75 standard deviations) of the mean and CoV of DNA intensities were calculated, and every object that exceeded either of these thresholds was scored as “mitotic.”

Microscopy data and image analysis scripts are available via the following Zenodo repositories:

Figure 1 and Figures 2, 3, and 4— https://doi.org/10.5281/zenodo.19473110 ,

Figure 4—https://doi.org/10.5281/zenodo.18436278 ,

Figure 5—https://doi.org/10.5281/zenodo.19472949

### 4.3 Drosophila Testes

#### 4.3.1 Drosophila Testes Sample Preparation

The housing and experimentation with fruit flies do not require individual and specific ethics approval and are exempt from such approval under the EU directive 2010/63/EU. Prior to sample preparation, a 96‐well plate with conical wells was prepared as followed: 4 wells were filled for each of the following solutions: PBS, 4% formaldehyde in PBS, 0.1% Triton X‐100 in PBS, PBS with 0.1% Tween‐20 (PBST) and 1% BSA in PBST. Flies were moved from a chosen vial into a new one. The old vial, containing pupae, but not adult flies, was incubated for 1 h at room temperature to obtain freshly hatched flies. Subsequently, the flies were anaesthetized using ${\mathrm{CO}}_{2}$ and transferred to a fly pad. The male flies were collected and transferred into a 500 μL drop of PBS on a dissection dish (black background). The flies were dissected to obtain testes, and 2–3 pairs of testes were gently placed into the first well filled with PBS. They were moved through four wells with PBS. Next, they were moved through all four wells with 4% formaldehyde to assure equilibration of the formaldehyde concentration, finally allowing for 20 min of fixation in the last formaldehyde well. Further, the testes were permeabilized by moving the samples through all four wells filled with 0.1% Triton X‐100 and an incubation step of 30 min in the last well with Triton X‐100. Lastly the samples were moved through the four wells filled with PBST and washed by being left in the last one for 15 min.

#### 4.3.2 Immunofluorescence

Immunostaining was performed directly after permeabilization. To block the samples for non‐specific antibody binding, the samples were moved through four wells of a 96‐well culture plate filled with 1% BSA in PBST and left to incubate for 45 min at room temperature in the last well. During this blocking procedure, a primary antibody solution (1:300 rabbit anti‐Pol II Ser2P and 1:300 rat anti‐Pol II Ser5P, or 1:300 rabbit anti‐H3K27ac and 1:300 rat anti‐Pol II Ser5P in 1% BSA in PBST, Table 3) was prepared and two remaining wells of the 96‐well plate were filled. The testes were transferred through the two wells of primary antibody solution and incubated in the second well in a moist dark chamber overnight at $4^{\circ }C$. The samples were moved through four wells with PBST and incubated in the last one for 15 min. The secondary antibodies solution (1:300 goat anti‐rabbit conjugated to Alexa 647 and 1:300 goat anti‐rat conjugated to Alexa 488 in 1% BSA in PBST, Table 3) was prepared and two wells were filled. The testes were moved through both wells with the secondary antibodies solution and incubated in the second well in a moist dark chamber overnight at $4^{\circ }C$. The samples were then moved through four wells with PBST for washing and incubated in the last one for 15 min. Finally, the testes were mounted on a poly‐L‐lysine slide in 30 μl Vectashield with 1:10 000 Hoechst 33342, covered with a #1.5 cover slip on top and sealed with nail polish.

#### 4.3.3 Microscopy

Microscopy data from *Drosophila melanogaster* testes were recorded using an LSM 900 confocal fluorescence microscope with Airyscan 2 with a Plan‐Apochromat 63×/1.40 Oil DIC M27 objective, either in confocal imaging mode for testis micrographs, or in super‐resolution imaging mode for quantification of the cells in the apical tip. In super‐resolution mode, a lateral resolution of ≈120nm and an axial resolution of ≈350nm can be achieved, which is similar to instant‐SIM. In a given experimental repeat, the illumination and acquisition settings were kept constant.

#### 4.3.4 Image analysis:

Nuclei and Pol II clusters were segmented based on the Pol II Ser5P channel by image processing steps that are identical to those used for mESCs. Parameter values were adjusted: nuclei segmentation foreground Gaussian kernel σ=0.6μm, nuclei background subtraction Gaussian kernel σ=8μm, nuclear segmentation mask erosion 0.1μm, topological hole‐filling in 2D, nuclei minimal volume $10\;\mu m^{3}$ and minimal solidity 0.4; cluster segmentation foreground Gaussian blur σ=0.001μm, cluster segmentation background subtraction Gaussian blur σ=0.1μm, robust background removal threshold 2 standard deviations above mean. DBSCAN ε=0.3μm, minimal cluster volume $0.005\;\mu m^{3}$. For the analysis underlying Figure 4C,D, additionally all objects greater than the nuclear maximum $10\;\mu m^{3}$ were removed during segmentation of nuclei.

To assign the segmented nuclei a coordinate that represents differentiation progress, the Hub‐SG border and the SG‐SpC border were marked manually with a point coordinate in each recorded image stack. Based on these two points, an axis could be digitally drawn into the image, allowing the projection of all the centroid xy‐positions of each nucleus to be projected to this axis. A projected value of 0 corresponds to the beginning of the differentiation process, a projected value of 1 to the completion of the differentiation process.

Similarly to the analysis of mESC image data, H3K27ac levels in images of fruit fly testes were corrected for differences in nuclear volume. Here, the volume of nuclei was used directly

(4) $$\begin{array}{c} l_{K27ac}=\frac{I_{K27ac}}{V}\frac{\tilde{V}}{{\tilde{I}}_{K27ac}}, \end{array}$$

where 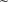 indicates the median over all nuclei.

Microscopy data and image analysis scripts underlying Figure 2 are available via the following Zenodo repository: https://doi.org/10.5281/zenodo.19473268.

Microscopy data and image analysis scripts underlying Figure 4C,D are available via the following Zenodo repository: https://doi.org/10.5281/zenodo.19455157.

### 4.4 Zebrafish Embryos

#### 4.4.1 Zebrafish Husbandry

The experiments with zebrafish embryos are concluded before 5 days after fertilization, requiring no specific animal experiment ethics approval under the applicable regulations. Approval is only required for the husbandry of wild‐type adult zebrafish: all zebrafish husbandry was performed in accordance with the EU directive 2010/63/EU and German animal protection standards (Tierschutzgesetz §11, Abs. 1, No. 1) and is under supervision of the government of Baden‐Württemberg, Regierungspräsidium Karlsruhe, Germany (Aktenzeichen35‐9185.64/BH KIT). Embryos used for the different experiments were obtained through spontaneous mating of adult wild‐type ABTL zebrafish. Collected embryos were dechorionated with Pronase, washed three times with E3 embryo medium, once with 0.3× Danieau's solution, and subsequently kept in agarose‐coated Petri dishes or 6‐well plates in 0.3× Danieau's solution at 28.5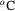.

#### 4.4.2 Sample Preparation

When zebrafish embryos reached chosen stages (oblong, sphere, dome, Perc% epiboly and 50% epiboly), five embryos each were transferred into 2 mL Eppendorf tubes filled with 1 mL embryo fixative (0.3x Danieau's, 2% formaldehyde (FA), 0.2% Tween‐20). For fixation the samples were left at $4^{\circ }C$ overnight. On the next day, the samples were washed three times with PBST for 5 min. Afterward, the embryos were transferred into a Petri dish filled with PBST and the animal caps of the embryos were carefully separated from the yolk by using two fine forceps. The animal caps were transferred back into a tube and either stored at $4^{\circ }C$ or were directly used. The animal caps were permeabilized with 1 ml permeabilization mix (PBS with 0.5% Triton X‐100) for precisely 15 min at room temperature. The samples were washed three times with PBST for 5 min. Finally the animal caps were directly used for immunostaining.

#### 4.4.3 Whole Zebrafish Embryo Inhibitor Treatment

Zebrafish embryos were treated with the chemical inhibitors flavopiridol (stock concentration 12.5 mM) or JQ1 (stock concentration 50 mM). In both cases, treatment proceeded for 30 min, starting such that 30 min of treatment finished when reaching sphere stage. Control embryos developed in parallel and were equally collected in sphere stage.

#### 4.4.4 Immunofluorescence

Directly after permeabilization the immunostaining was carried out. The samples were blocked with 4% BSA in PBST for 30 min at room temperature. Depending on the experimental setup, primary antibodies (anti‐H3K27ac, anti‐Pol II Ser2P and anti‐Pol II Ser5P in 4% BSA in PBST) were applied overnight at $4^{\circ }C$. The samples were washed three times with PBST for 5 min. The samples were washed once with 4% BSA in PBST. Depending on the experiment, different secondary antibodies were used (for details on antibodies, see Table 3). The secondary antibodies were applied in 4% BSA in PBST overnight at $4^{\circ }C$. The samples were washed three times with PBST for 5 min. For the two‐color immunofluorescence of Pol II Ser5P and Pol II Ser2P, a previously established and validated antibody combination was used (primary antibodies: 1:300 rabbit anti‐Pol II Ser2P, ab193468; 1:300 rat anti‐Pol II Ser5P in 4% BSA in PBST; secondary antibodies: 1:300 anti‐rat IgG Alexa 594 and 1:300 anti‐rabbit IgG Alexa 488 in 4% BSA in PBST) [19]. PBST was carefully removed from the animal caps of the embryos under the microscope to prevent damaging or losing the animal caps. For mounting 30 μl of Vectashield H‐1000 with 5μM Hoechst 33342 (1:2,500 dilution from a 20 mM stock concentration) was added to every sample. The animal caps were transferred with a P20 micropipette on a microscope slide. The slide was covered with a cover slip (#1.5, selected) and the slides were sealed with nail polish.

For the three‐color immunofluorescence of Pol II Ser5P, Pol II Ser2P, and H3K27ac, a mouse IgM and a rat IgG primary antibody had to be used, introducing a small risk of crosstalk in the secondary antibody staining step. To reduce this risk of crosstalk, two rounds of indirect immunofluorescence were carried out. First, a mouse IgM primary antibody (1:300 anti‐Pol II Ser2P in 4% BSA‐PBST, see Table 3) was incubated overnight at 4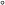, then an anti‐mouse IgM secondary antibody (1:300, anti‐mouse IgM, conjugated to Alexa 594). Subsequently, a rat IgG and a rabbit IgG primary antibody (1:300 rabbit anti‐H3K27ac and 1:300 rat anti‐Pol II Ser5P) were incubated overnight, followed by overnight incubation of according secondary antibodies (1:300 anti‐rabbit conjugated to Alexa 488 and 1:300 anti‐rat conjugated to Alexa 647). The potential crosstalk from the mouse IgM‐based detection into the rat IgG‐based detection was not apparent (Figure S9). Raw data and example images for crosstalk assessment are available via the following Zenodo repository: https://doi.org/10.5281/zenodo.8031698.

#### 4.4.5 Microscopy

Microscopy data from Zebrafish embryos were recorded using a VisiTech iSIM high‐speed super‐resolution confocal microscope as described for the recording from mESCs.

#### 4.4.6 Image Analysis

The segmentation of nuclei and Pol II clusters was based on the DNA and Pol II Ser5P channels, respectively, and was implemented in a pipeline with steps identical to the image processing for mESCs and sperm precursor cells. For the assessment of Pol II clusters in different stages (Figure 3), parameter values were adjusted: nuclei segmentation foreground Gaussian kernel σ=3μm, nuclei background subtraction Gaussian kernel σ=10μm, nuclear segmentation mask erosion 1.5μm, topological hole‐filling in 3D, nuclei minimal volume $40\;\mu m^{3}$, maximal volume $500\;\mu m^{3}$, and minimal solidity 0.8; cluster segmentation foreground Gaussian blur σ=0.001μm, cluster segmentation background subtraction Gaussian blur σ=0.1μm, robust background removal threshold 1.5 standard deviations above mean. Minimal cluster volume $0.1\;\mu m^{3}$ (Figure 3) or $0.01\;\mu m^{3}$ (Figure 5), DBSCAN ε=0.65μm. The raw image data and analysis scripts are provided via the following Zenodo repository: https://doi.org/10.5281/zenodo.19472996.

For the assessment of H3K27ac levels at Pol II clusters in different stages (Figure 4), Pol II Ser5P signal was used for segmentation of nuclei as well as Pol II clusters. Parameter values were adjusted: nuclei segmentation foreground Gaussian kernel σ=3.0μm, nuclei background subtraction Gaussian kernel σ=10μm, nuclear segmentation mask erosion 1.5μm, topological hole‐filling in 3D, nuclei minimal volume $10\;\mu m^{3}$ and minimal solidity 0.8; cytoplasmic mask reaching 1.0−1.5μm, cluster segmentation foreground Gaussian blur σ=0.001μm, cluster segmentation background subtraction Gaussian blur σ=0.1μm, robust background removal threshold 1.5 standard deviations above mean. Minimal cluster volume $0.1\;\mu m^{3}$, DBSCAN ε=0.65μm. The raw image data and analysis scripts are provided via the following Zenodo repository: https://doi.org/10.5281/zenodo.18436671

For the assessment of Pol II clusters upon flavopiridol and JQ1 treatment (Figure 6), nuclei were segmented based on DNA signal and Pol II clusters based on Pol II Ser5P signal. Parameter values were adjusted: nuclei segmentation foreground Gaussian kernel σ=3μm, nuclei background subtraction Gaussian kernel σ=10μm, nuclear segmentation mask erosion 1.5μm, topological hole‐filling in 3D, nuclei minimal volume $10\;\mu m^{3}$ and minimal solidity 0.8; cytoplasmic mask reaching 1.0−1.5μm, cluster segmentation foreground Gaussian blur σ=0.001μm, cluster segmentation background subtraction Gaussian blur σ=0.1μm, robust background removal threshold 1.5 standard deviations above mean. Minimal cluster volume $0.01\;\mu m^{3}$, DBSCAN ε=0.65μm. The raw image data and analysis scripts are provided via the following Zenodo repository: https://doi.org/10.5281/zenodo.18436602

Normalization of H3K27ac levels for nuclear volume differences was applied identically to the analysis of mESC image data.

### 4.5 Biophysical Lattice Model for Cluster Description

For the theoretical biophysical description of the experimentally observed phenomena, we use our existing lattice kinetic Monte‐Carlo (LKMC) model of surface condensation [19]. While all parts that are essential to reproduce our results are described in here, the mentioned publication and publicly hosted simulation scripts can be used as reference for a more detailed description of the model.

#### 4.5.1 Model Components

The model consists of four different species (see Figure S10A). First, liquid material containing recruited RNA Pol II (S5P, red), the species that actually forms clusters, is represented by single lattice sites. In addition, a polymer that serves as surface for cluster formation is modeled as a chain of connected lattice sites. To represent different activities and functions of chromatin, the polymer consists of three different subregions, lined up in the following order: inactive chromatin (IC, black), regulatory chromatin (RC, blue) and active chromatin (AC, gray), and flanked again by inactive chromatin.

#### 4.5.2 Inter‐ and Intraspecies Affinities

To enable formation of clusters, self affinity ($w_{S5P-S5P}<0$) is assumed (see Figure S10B). The parameter is adjusted so that canonical phase separation does not happen. This is the case for all chosen values $w_{S5P-S5P}\in \left[-0.2,-0.25,-0.35\right]$. The different polymer subregions have different affinities to S5P and with themselves. IC is subject to self affinity ($w_{IC-IC}<0$), reflecting a tendency for compaction, and is neutral to other chromatin species and S5P. RC has no self affinity, but instead affinity to S5P ($w_{RC-S5P}<0$). AC and S5P repel each other ($w_{AC-S5P}>0$), as it was shown that elongating Pol II is excluded from transcriptional clusters.

#### 4.5.3 Move Set

S5P is implemented as single particles within the lattice and can move to all eight nearest neighbor lattice sites (see Figure S10C). Movements are only prohibited in the case an S5P particle is located at the lattice border, since movements outside the lattice boundaries are not permitted, or in the case, the targeted neighboring site is already occupied by another S5P particle. For the polymer, the known Verdier–Stockmayer move set, consisting of kink‐jump, end‐bond flip and crankshaft move, is used [82, 114, 115]. This restricted move set allows simulation of self‐avoiding polymers. S5P and polymers have no space exclusion interactions and evolve on different lattice layers.

#### 4.5.4 Initial Configuration

All simulations are performed within a lattice of same size (25×25 lattice sites) (see Figure S10D). The numbers of S5P ($N_{S5P}=100$) and polymers ($N_{Polymer}=4$) are kept constant for all simulations. The single S5P particles are randomly distributed within the lattice at the beginning of a given simulation. Polymers overall have the same length ($L_{Polymer}=20$), whereas the length of each subregion ($N_{IC}$, $N_{RC}$, and $N_{AC}$) is varied for different developmental stages or treatments. $N_{RC}\in \left[0,2,4,6,8\right]$ (per polymer), $N_{AC}\in \left[0,3,6\right]$ (per polymer), $N_{IC}$ as much as necessary to fill up to the total length $L_{Polymer}$. At the beginning of the simulation, four polymer chains are stacked vertically in the center of the lattice and their orientation is alternated.

#### 4.5.5 Simulation Framework

The coarse‐grained LKMC simulations [82, 116] act as a Gillespie‐type algorithm [117] and run rejection‐free (see Figure S10E). After setting the initial configuration at the beginning of a given simulation, all lattice sites are checked for possible system transitions. These transitions are then saved together with their rates k (determined by energy difference and move type) and the total system energy $k_{total}$ as sum of all transition rates. Out of this catalog, one transition is chosen by the tower sampling method, which takes into account a random number r∈[0,1] and the system energy. This transition is then performed, immediately followed by a local update of the affected positions and proceeding to choosing the next transition. The choosing and performing loop is repeated N times. Most simulations were performed with a total of $1\times 10^{6}$ LKMC iteration steps.

#### 4.5.6 Simulation Analysis

To be able to compare the simulation output to experimental results and use the same image analysis pipeline, synthetic microscopy images are produced (see Figure S10F). To this end, we blur the images produced by the lattice model with Gaussian kernel (σ=1) and add Poisson‐distributed random numbers (λ=5, resulting random numbers divided by 100) to simulate detector noise. For cluster detection, blurred distributions of S5P are used, to which a global segmentation threshold of 0.35 is applied, cluster labeling (connected‐component labeling, CCL) is performed, and labeled clusters are retained if they exceed a minimal area of 10. We used ergodic sampling (every $1\times 10^{4}$ step) for simulation snapshots after $8\times 10^{5}$ initial time steps for equilibration. For each of these sampling time points, cluster area, solidity, and S5P and S2P intensities are measured. For the image analysis of inhibitor simulations, we applied a 2D Gaussian KDE, so that for each detected cluster the solidity and area values contributed to a smooth density, which we converted into a matrix with probability density values by multiplying with the bin cell area and normalizing. For the Control case, we visualized the resulting probability distribution $P_{Control}\left(x,y\right)$, while for Flavopiridol and JQ‐1 we displayed the corresponding difference maps $\Delta P_{Flavopiridol}=P_{Flavopiridol}\left(x,y\right)-P_{Control}\left(x,y\right)$ and $\Delta P_{JQ1}=P_{JQ1}\left(x,y\right)-P_{Control}\left(x,y\right)$. This yielded KDE‐based estimates of the probability distributions of the inhibitor conditions. The numerical simulations as well as the image analysis are carried out using Python (Spyder).

Data sets and scripts for theoretical model are available via the following Zenodo repository: https://doi.org/10.5281/zenodo.18392762.

## Author Contributions

Conceptualization: TK, IW, AP, SE, VZ, CF, LH. Investigation: TK, IW, AP, MA, YG, PK, AMF, YB, ASH, MP, AG, MS, EK, SEK, SA, CF, LH. Writing – original draft: TK, IW, AP, VZ, CF, LH. Writing – review & editing: TK, IW, AP, SA, SE, VZ, CF, LH. Software: TK, SA, LH. Supervision: SE, VZ, CF, LH. Funding acquisition: SA, SE, VZ, CF, LH

## Conflicts of Interest

The authors declare that they have no conflicts of interest.

## Use of Artificial Intelligence

The artificial intelligence tools Perplexity, Consensus, and Claude were used for literature search, refining grammar and expression, and for editing the Introduction section. All text was reviewed and edited in final form by the human authors, who take full responsibility.

## Supporting information


**Supporting File**: advs75924‐sup‐0001‐SuppMat.pdf.

## Data Availability

Microscopy data, image analysis scripts, and simulation scripts underlying this study are publicly accessible online without any restrictions via the Zenodo repositories referenced in the Methods section.
