# Supplementary material for: Stem Cell Differentiation Disperses Transcriptional Clusters via a Conserved Surface‐Condensate Trajectory
Source: Adv Sci (Weinh). 2026 Jun 9;13(49):e75924. doi: 10.1002/advs.75924 (PMC13336672; doi:10.1002/advs.75924)
Supplement: Supplementary file 1 [file ADVS-13-e75924-s001.pdf]

WILEY-VCH

Stem cell differentiation disperses transcriptional clusters via a conserved surface-condensate trajectory

Tim Klingberg Irina Wachter Agnieszka Pancholi Matthias Akyel Yomna Gohar Priya Kumar Ana Miguel Fernandes Yuzhi Bao Alica Schmidt-Heydt Marcel Piepers Alicia Günthel Marcel Sobucki Elisa Kämmer Süheyla Eroğlu-Kayıkçı Stephan Allgeier Sylvia Erhardt Vasily Zaburdaev Carmelo Ferrai Lennart Hilbert

Supplementary Figures

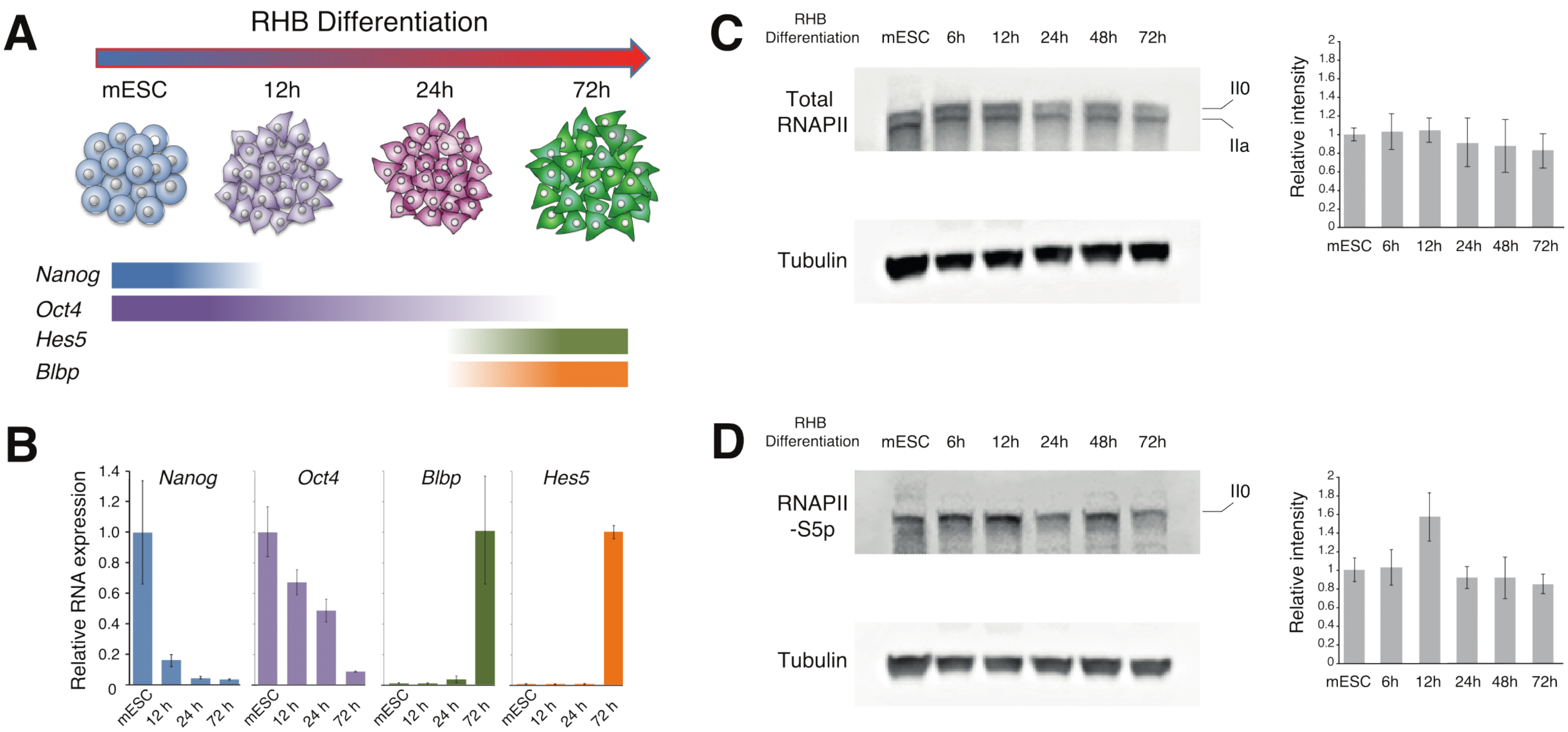

Figure S1: **Mouse embryonic stem cell (mESC) pluripotency exit induces a transient increase in RNA polymerase II C-terminal repeat Serine 5 phosphorylation.** A) Schematic representation of the RHB differentiation system and the temporal expression dynamics of differentiation stage marker genes. B) RNA levels of differentiation stage markers were measured by qRT-PCR. Relative levels are normalized to β-Actin (Actb) internal control and values are plotted as fold change relative to the highest expressed time point. Values are mean $\pm$ standard deviation from three biological replicates. C) Total RNA polymerase II levels were assessed by Western blot. Total RNA polymerase II band intensities were normalized against Tubulin loading controls and values are plotted relative to the undifferentiated timepoint (marked as "mESC"). Values are mean $\pm$ standard deviation from four biological replicates. D) The levels of Serine 5 phosphorylation of RNA polymerase subunit 1 C-terminal heptad repeat (RNAPII-S5p) were assessed by Western blot. RNAPII-S5p band intensities were normalized against tubulin loading controls and values are plotted as fold changes relative to the undifferentiated time point (marked as "mESC"). Vales are mean $\pm$ standard deviation from four biological replicates.

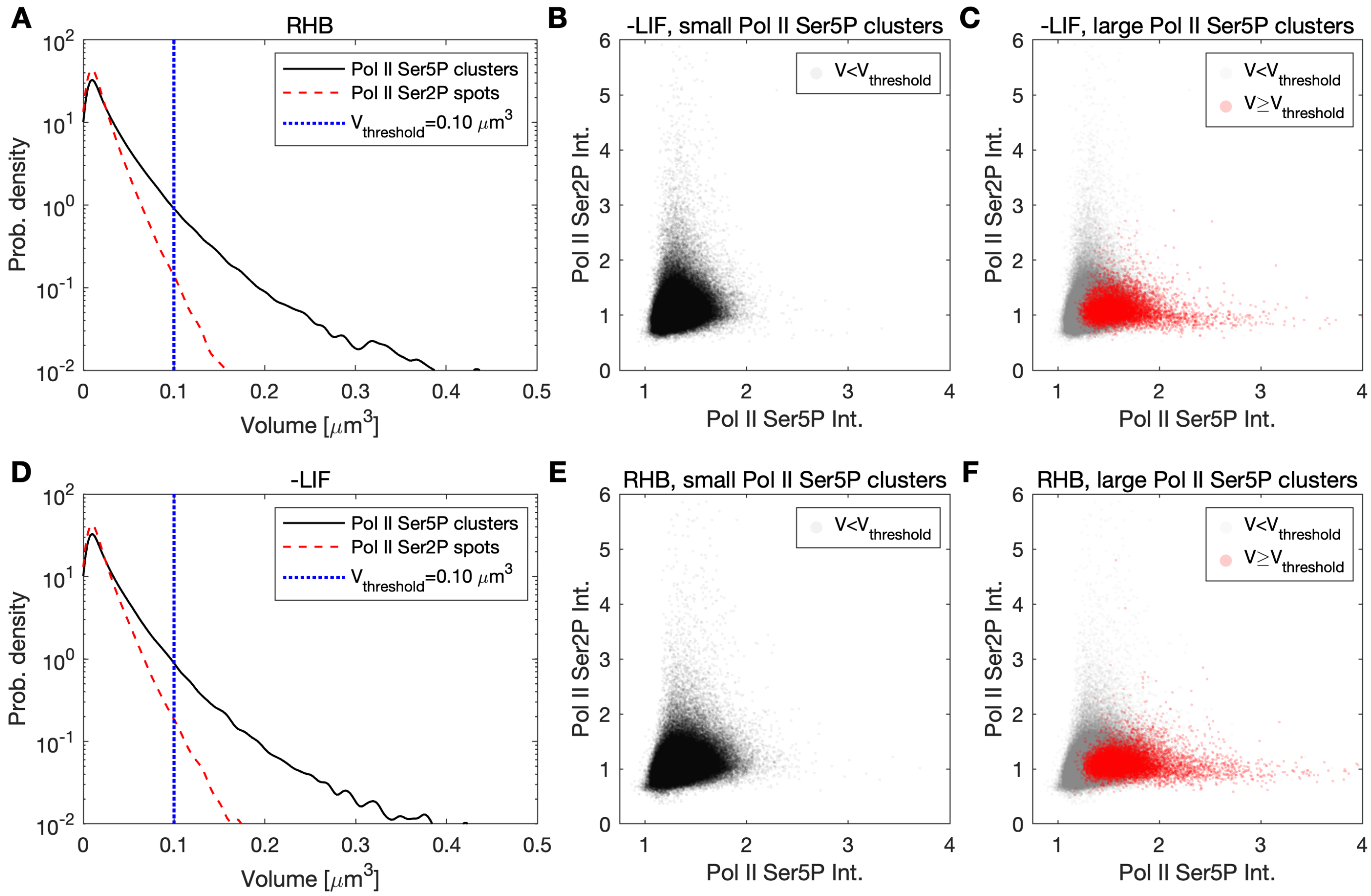

Figure S2: **Setting a robust volume threshold to select only large RNA polymerase II clusters for further analysis. A)** Distribution of volumes for clusters of recruited RNA polymerase II (labeled via Pol II Ser5P immunofluorescence) and spots of elongating RNA polymerase II (labeled via Pol II Ser2P immunofluorescence) in mouse embryonic stem cells (mESC) undergoing RHB differentiation. Probability densities are empirically determined by Gaussian kernel-based smoothing. Above a volume cut-off threshold $V_{threshold} \ = \ 1.0\,\mu\text{m}^3$, a robustly higher frequency of large clusters of recruited Pol II than of spots of elongating Pol II is seen. Data from all differentiation time points were pooled. **B, C)** Two-dimensional scatter plots of Pol II Ser5P and Pol II Ser2P intensity levels of small (B) and large-plus-small (C) Pol II Ser5P clusters were used to validate the chosen volume threshold. The overlaid display matches the intensity distributions of small and large clusters established in previous work using super-resolution microscopy [1], indicating a valid choice of volume threshold. **D)** Distribution of cluster and spot volumes for mESC undergoing LIF-withdrawal-induced differentiation, data pooled from all differentiation time points. **E, F)** Same as B and C, but for LIF-withdrawal-induced differentiation.

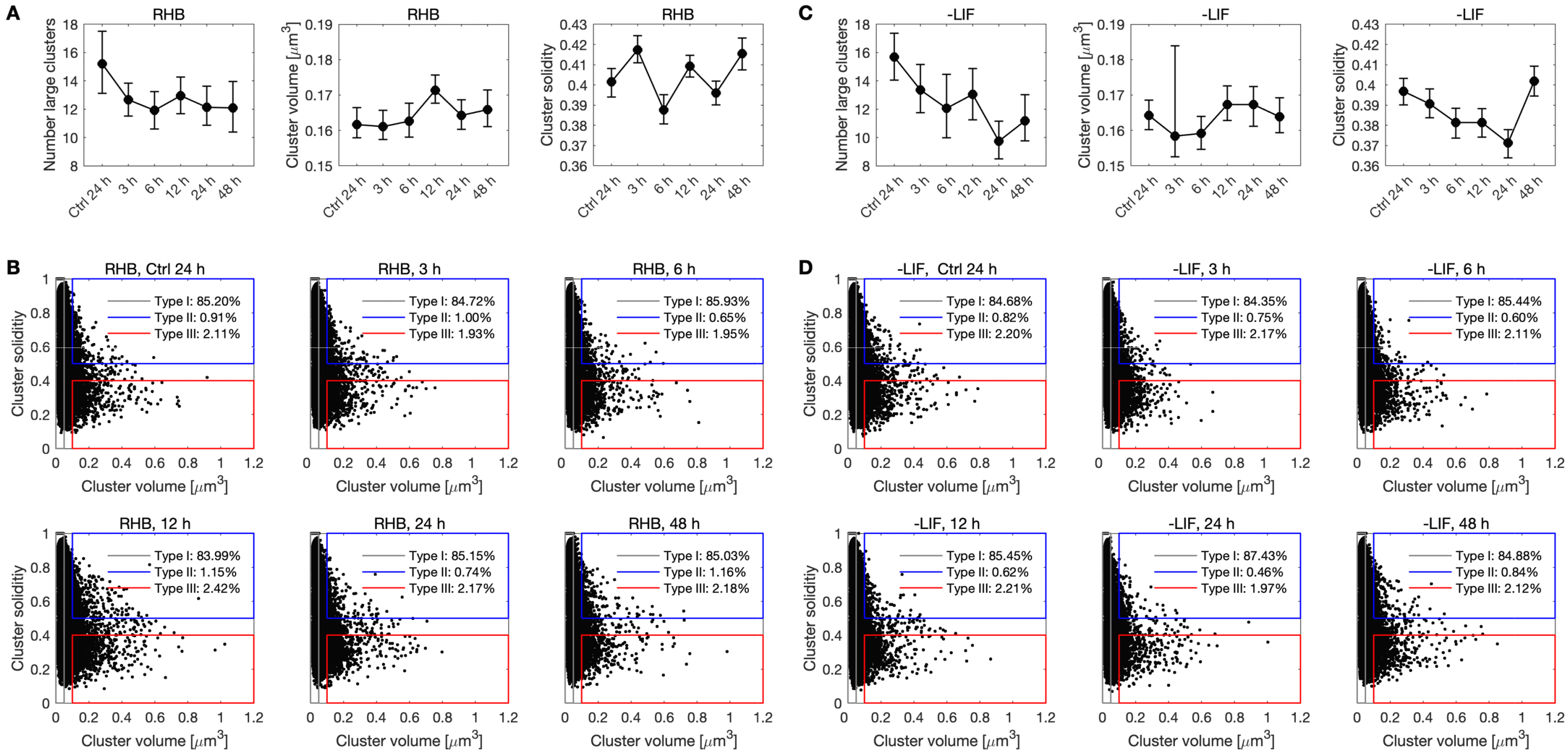

Figure S3: **Analysis of cluster properties per time point of mESC differentiation protocols. A)** Number of large clusters of recruited Pol II (cluster volume of at least $0.1\,\mu\text{m}^3$), volume of large clusters, and solidity of large clusters for all time points of RHB differentiation. Mean values with 95% bootstrap confidence intervals (1000 times bootstrap resampling). **B)** Volume-solidity scatter plot of all clusters of recruited Pol II for all time points of RHB differentiation. Gates indicate cluster morphology types in line with previous work: small (I), large and rounded (II), and large and unfolded (III) [1]. For each of the corresponding gates, the percentage of all clusters detected in this gate are displayed. Number of clusters in each of the time points: 41266, 38862, 35717, 50786, 44336, 28703. **C)** Same as A, but for -LIF differentiation. **D)** Same as B, but for -LIF differentiation. Number of clusters in each of the time points: 39827, 32953, 31014, 33619, 36777, 34712.

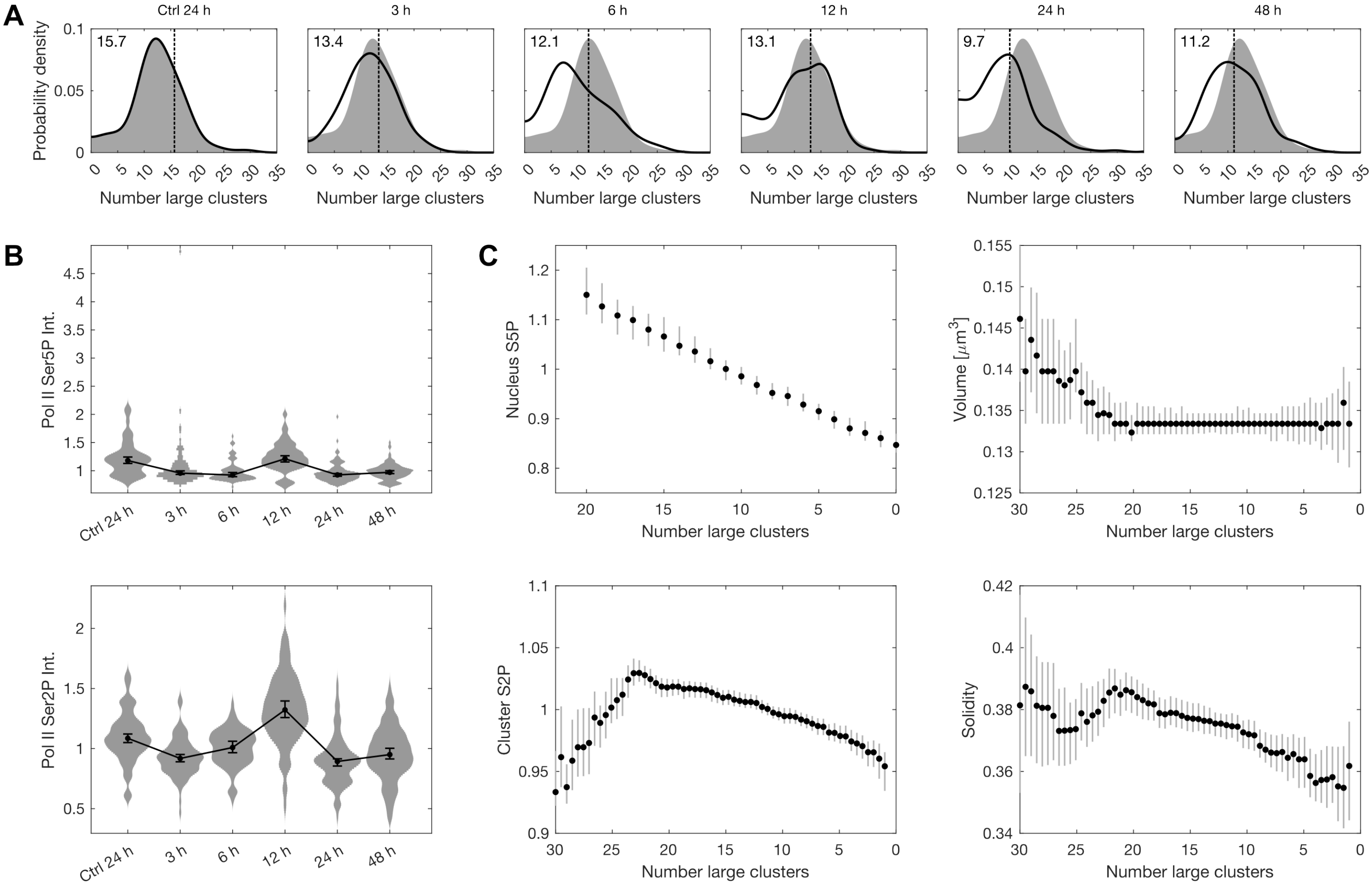

Figure S4: **Formation and dispersal of RNA polymerase II (Pol II) clusters in mouse embryonic stem cells upon leukaemia inhibitory factor (LIF) withdrawal. A)** Clusters per nucleus, kernel-based empirical probability density distribution, dashed line and text indicating arithmetic mean. In the different conditions, $n = 101, 94, 91, 95, 116, 120$ nuclei from two independent experimental repeats were detected. **B)** Whole-nucleus immunofluorescence intensities, normalized against the median of all values, data points are median with 95% confidence interval). **C)** Nuclei are binned based on a sliding window for the number of large clusters per nucleus and all large Pol II Ser5P clusters ($V \geq 0.1\,\mu m$) from all nuclei in a given window are used to calculate for a given bin the nuclear Pol II Ser5P and Pol II Ser2P intensity, cluster volume, and solidity of the cluster (median with 95% bootstrap confidence interval). 617 nuclei included in total. Number of included nuclei from each condition as above. 7647 clusters included in total (cluster volume threshold $0.1\,\mu m$). Running window with median and 95% bootstrap confidence interval, 1000 bootstrap resamples. Running window parameters: window width 12, minimal number of data points 12. Note that, in the analysis of cluster volumes, the large confidence intervals above $\approx$ 27 large clusters per nucleus indicate that an interpretation of differences in this range is not statistically warranted.

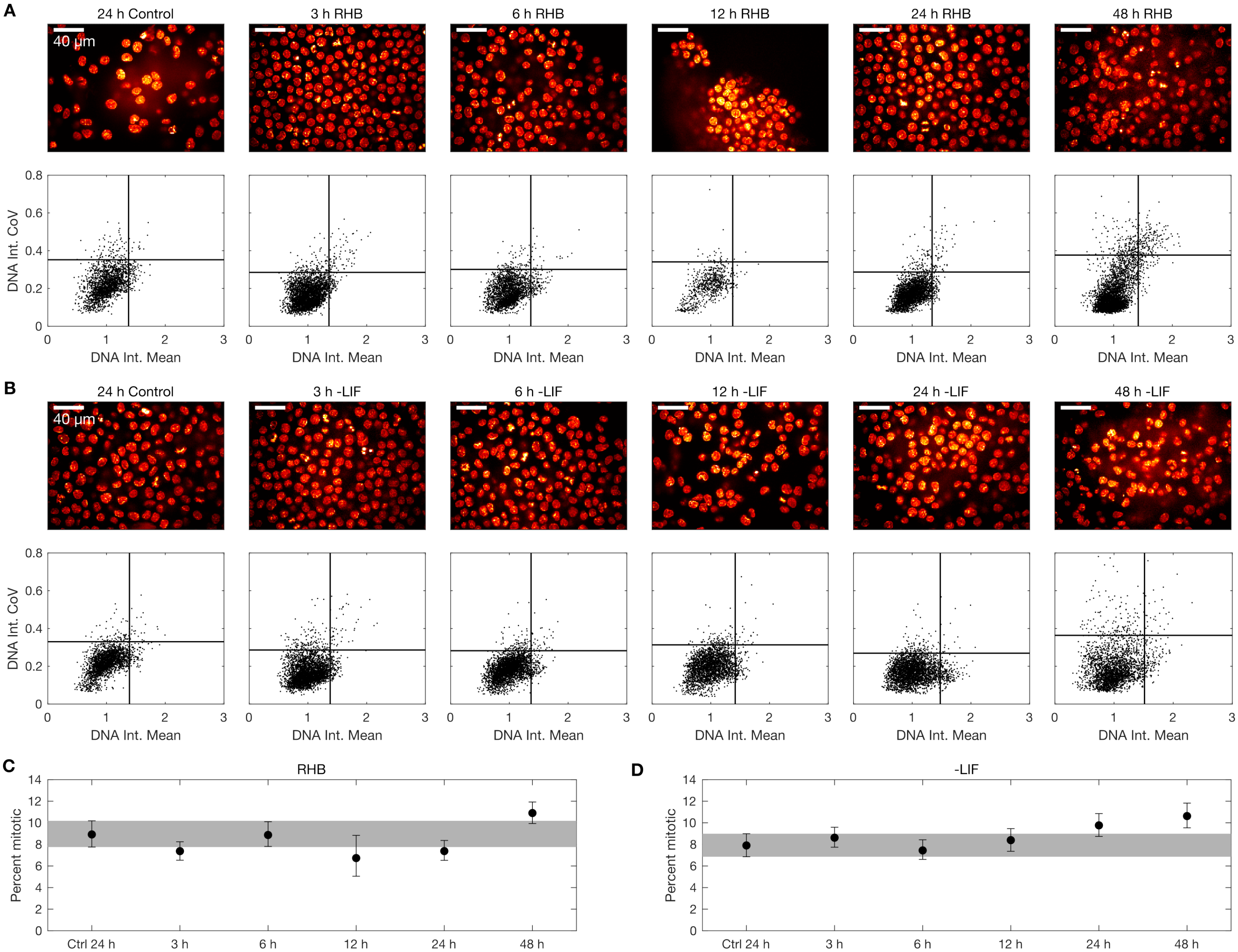

Figure S5: **Differentiation of mouse embryonic stem cells does not result in obvious cell cycle synchronization. A)** RHB, example micrographs are confocal sections of suspension-cultured cells, fixed and then mounted in liquid mounting media. Differences in cell density result from staining and mounting procedures, not from differences in culturing. n=2141, 3458, 2446, 713, 3036, 3916 nuclei, from two independent experiments. Recorded with 40X water immersion objective on instant-SIM. **B)** -LIF from n=2392, 3515, 3252, 2717, 3124, 2664 nuclei, from two independent experimental repeats. **C, D)** Percentage of objects scored as mitotic, mean with 95% boostrap confidence intervals, 5000 random draws per condition. The gray region marks the control condition confidence interval.

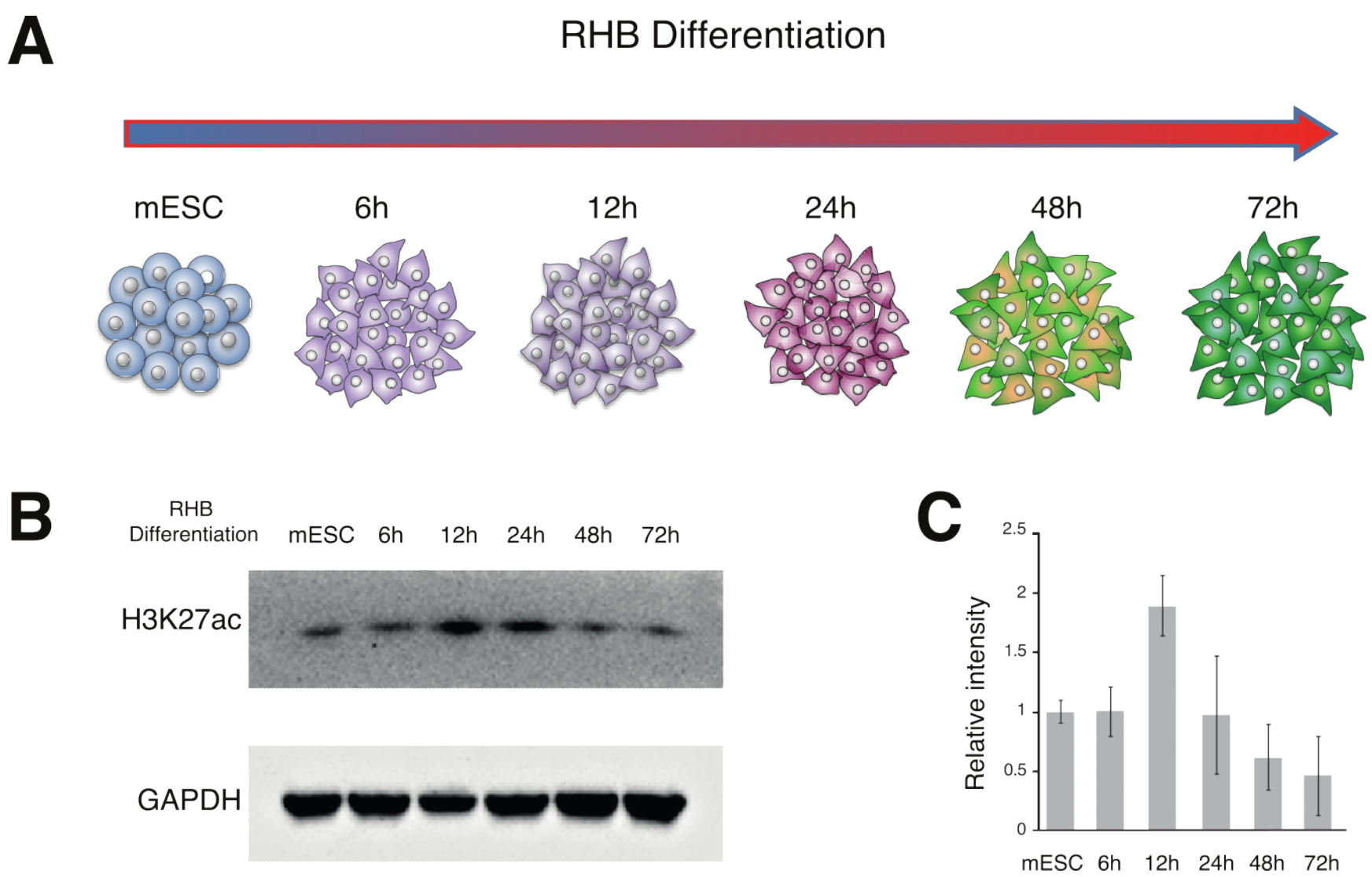

Figure S6: **Pluripotency exit induces a transient increase in H3K27ac. A)** Schematic representation of the mESC RHB differentiation system and the temporal expression dynamics of the differentiation stage collected time points **B)** H3K27ac levels during mESC RHB differentiation were assessed by Western blot. **C)** H3K27ac band intensities during mESC RHB differentiation were normalized against GAPDH loading controls and values are plotted relative to the undifferentiated time point (marked as "mESC"). Values are mean±standard deviation from three biological replicates.

A

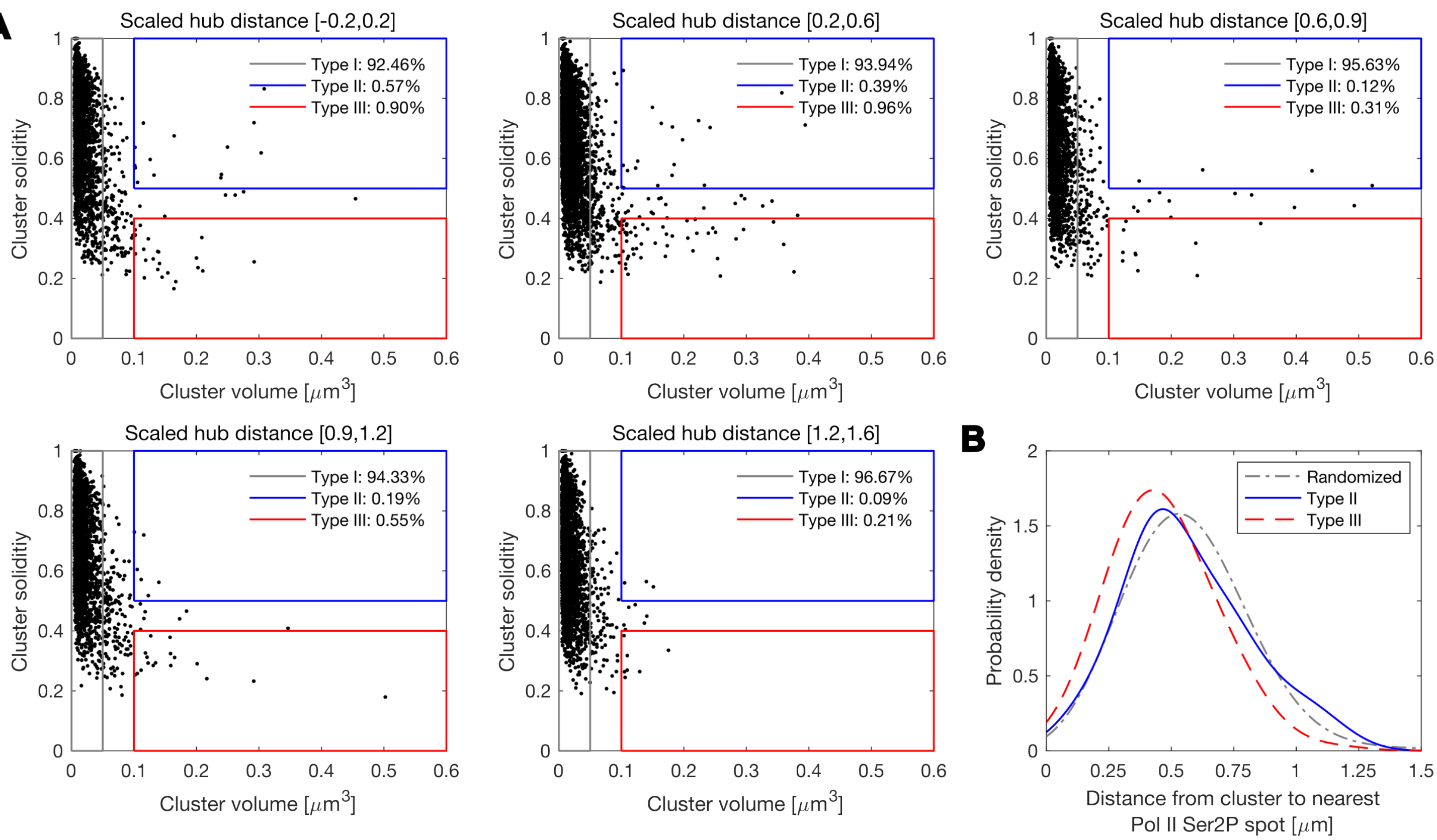

Figure S7: **Analysis of cluster properties and placement of elongating Pol II spots relative to large clusters in fruit fly testes. A)** Volume-solidity scatter plot of all clusters of recruited Pol II within the indicated scaled hub distance coordinates. Gates indicate cluster morphology types in line with previous work: small (I), large and rounded (II), and large and unfolded (III) [1]. For each of the corresponding gates, the percentage of all clusters detected in this gate are displayed. Number of clusters analyzed in each of the scaled hub distance ranges: 2440, 4898, 3272, 3085, 3360. **B)** Distribution of the distance of the nearest elongating Pol II (Pol II Ser2P) spot from the center of mass of a given large cluster ($V \geq 0.1\mu\text{m}^3$) of recruited Pol II (Pol II Ser5P). Clusters are pooled over all scaled hub distances. Type II and type III clusters are gated as indicated in panel A. For the Randomized reference curve, the number concentration of elongating Pol II clusters was calculated from image data and a random distribution of points at equal concentration was prepared for distance estimation. The probability densities are Gaussian kernel-based empirical estimates, kernel bandwidth $\sigma = 0.125\,\mu\text{m}$.

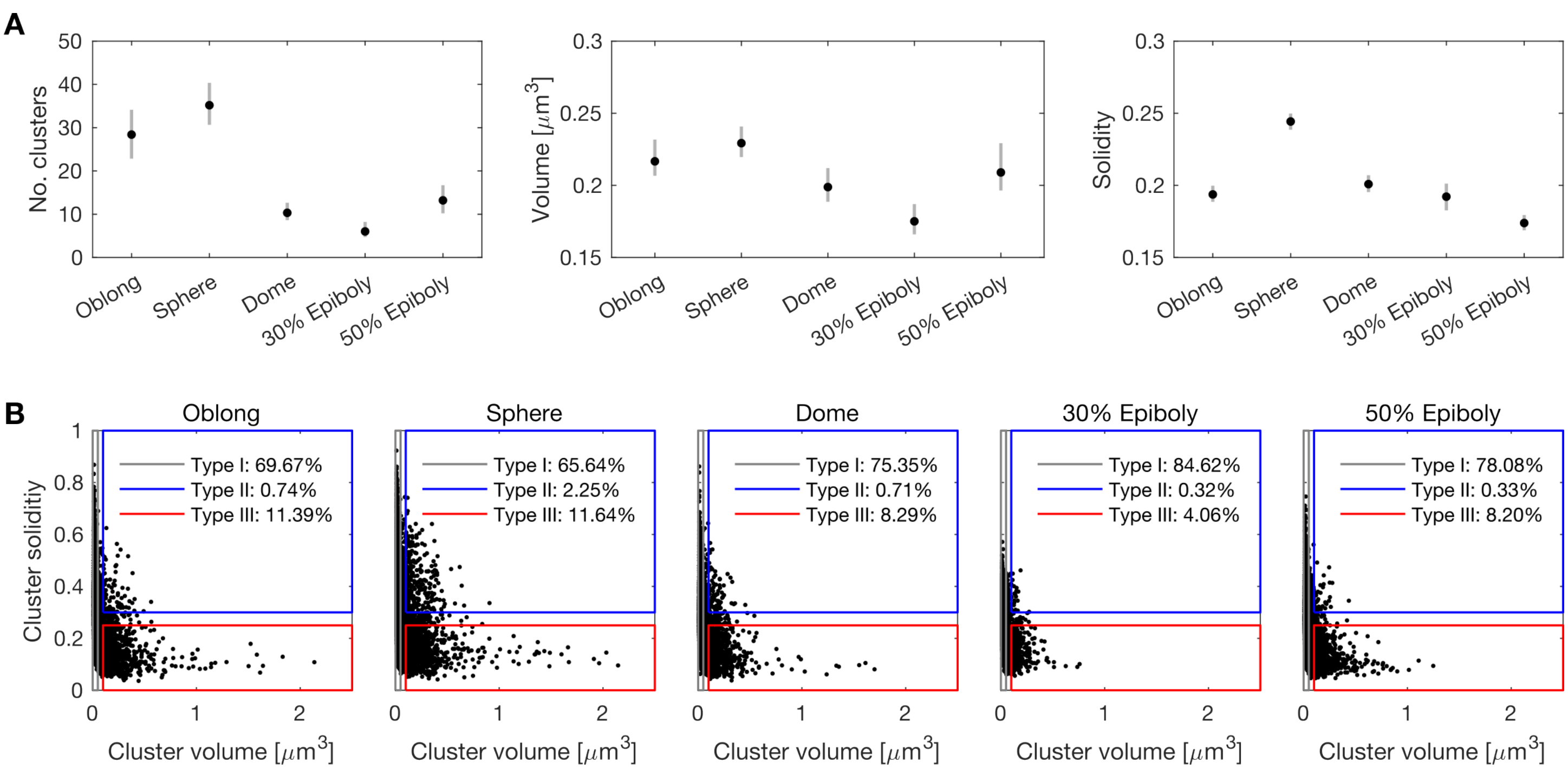

Figure S8: **Analysis of cluster properties per stages of zebrafish embryo development.** A) Number of large clusters of recruited Pol II (cluster volume of at least $0.1\,\mu m^3$), volume of large clusters, and solidity of large clusters for the five developmental stages of zebrafish development assessed in this study. Mean values with 95% bootstrap confidence intervals (1000 times bootstrap resampling). B) Volume-solidity scatter plot of all clusters of recruited Pol II for the five developmental stages. Gates indicate cluster morphology types in line with previous work: small (I), large and rounded (II), and large and unfolded (III) [1]. For each of the corresponding gates, the percentage of all clusters detected in this gate are displayed. Number of clusters analyzed in each of the consecutive stages: 11496, 12151, 10562, 7269, 11157 clusters.

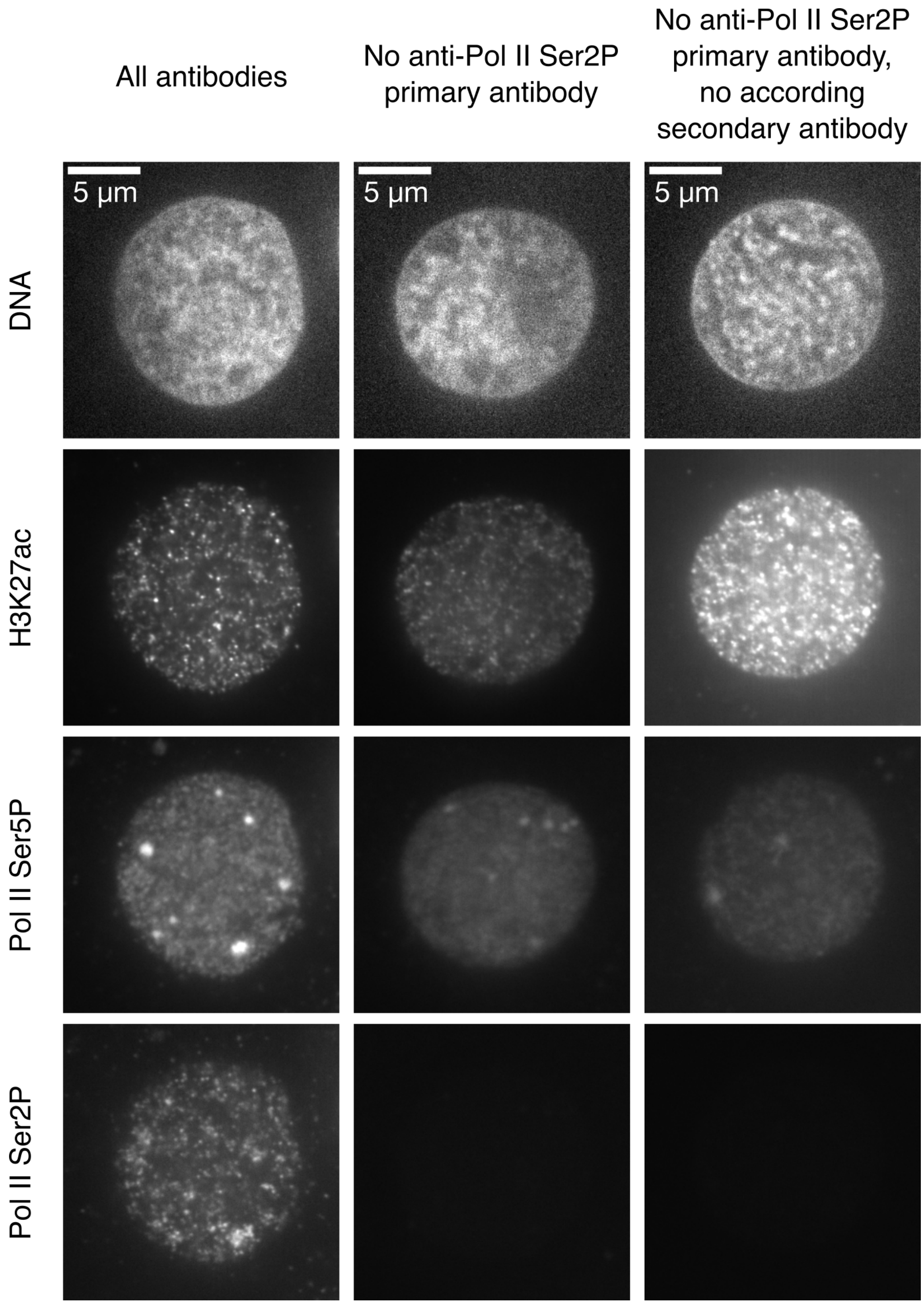

Figure S9: **Detection of elongating RNA polymerase II by a mouse IgM antibody exhibits no obvious cross-talk from the detection of recruited RNA polymerase II by a rat IgG antibody.** The staining protocol and image acquisition procedure were kept constant across all samples. Look-up tables for image display are identical for all immunofluorescence channels for direct comparison of intensities. Similar results were observed for $N = 2, 3, 3$ embryos per condition, viewing $n = 18, 30, 30$ different positions in these embryos.

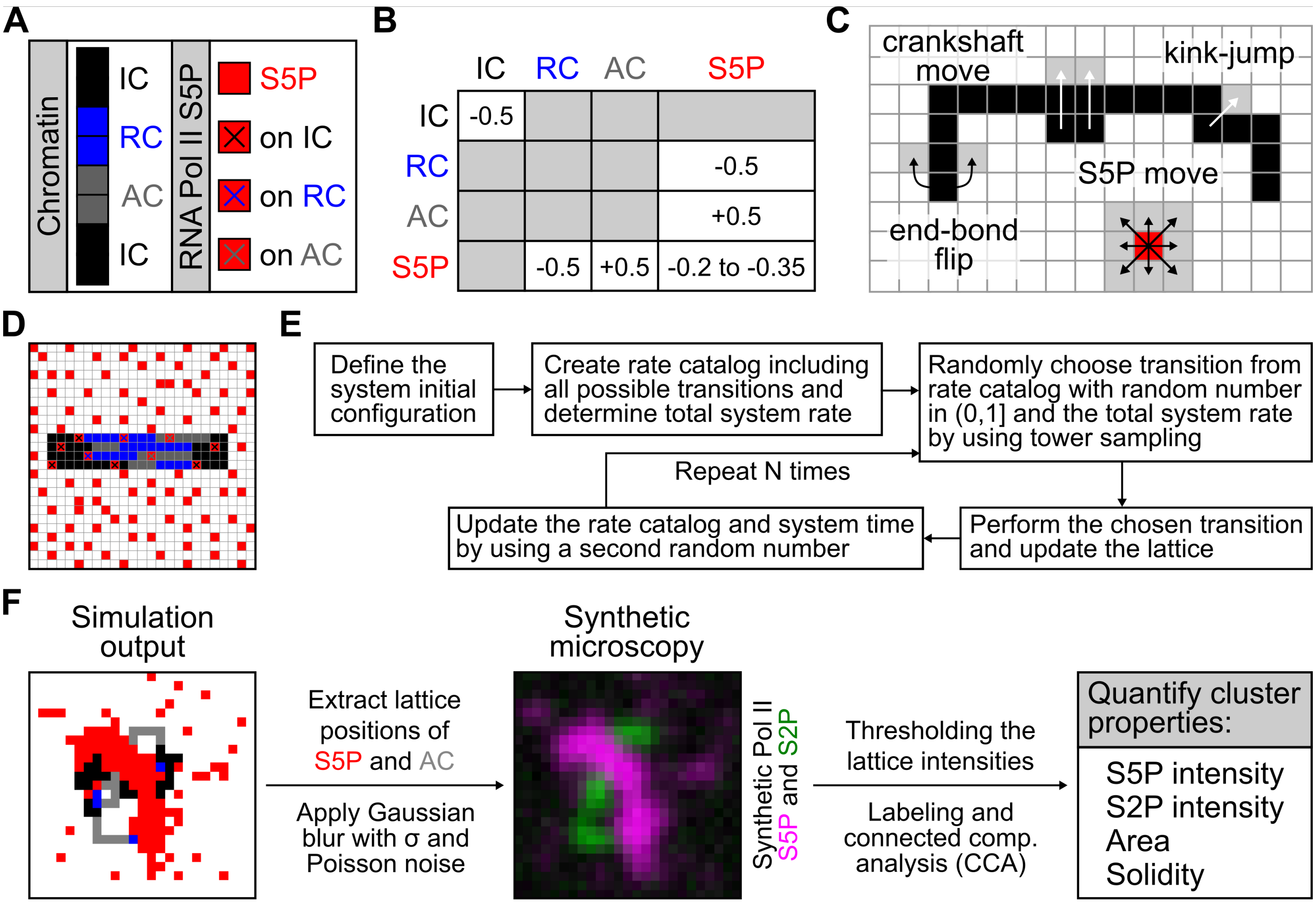

Figure S10: **Theoretical model of surface condensation. A)** Model components: Chromatin as polymer with different subregions and S5P as single lattice sites. **B)** Interspecies affinities: Negative values are attraction, positive ones repulsion and gray sectors are neutral. **C)** Move set: Verdier-Stockmayer move set for polymers, including end-bond flip, kink-jump and crankshaft move. Single S5P lattice sites can move to all eight nearest neighbouring sites. **D)** Lattice initial configuration: Stack of four polymers ($N_{Polymer} = 4$) of length $L_{Polymer} = 20$ within a 25×25 lattice. Single S5P ($N_{S5P} = 100$) are randomly distributed within the lattice. **E)** General simulation framework for the lattice kinetic Monte-Carlo (LKMC) simulations. **F)** Analysis pipeline of simulation results including production of synthetic microscopy images and quantification of cluster properties.

References

[1] Agnieszka Pancholi, Tim Klingberg, Weichun Zhang, Roshan Prizak, Irina Mamontova, Amra Noa, Marcel Sobucki, Andrei Yu Kobitski, Gerd U Nienhaus, Vasily Zaburdaev, and Lennart Hilbert. RNA polymerase II clusters form in line with surface condensation on regulatory chromatin. *Molecular Systems Biology*, 17(9):e10272, 2021.
